# Small protein blockers of human IL-6 receptor alpha inhibit proliferation and migration of cancer cells

**DOI:** 10.1186/s12964-024-01630-w

**Published:** 2024-05-07

**Authors:** Yaroslava Groza, Lukáš Lacina, Milan Kuchař, Leona Rašková Kafková, Kateřina Zachová, Olga Janoušková, Radim Osička, Jiří Černý, Hana Petroková, Joanna Maria Mierzwicka, Natalya Panova, Petr Kosztyu, Kristýna Sloupenská, Jan Malý, Jozef Škarda, Milan Raška, Karel Smetana, Petr Malý

**Affiliations:** 1 Laboratory of Ligand Engineering, Institute of Biotechnology of the Czech Academy of Sciences, BIOCEV Research Center, Prumyslova 595, Vestec, 252 50 Czech Republic; 2 Institute of Anatomy, 1st Faculty of Medicine, Charles University, U Nemocnice 3, Prague 2, 12800 Czech Republic; 3 Department of Dermatovenerology, 1st Faculty of Medicine, Charles University, U Nemocnice 2, Prague 2, 12000 Czech Republic; 4 Department of Immunology, Faculty of Medicine and Dentistry, Palacky University Olomouc and University Hospital Olomouc, Hněvotínská 3, Olomouc, 779 00 Czech Republic; 5 Centre of Nanomaterials and Biotechnologies, University of J. E. Purkyně in Ústí nad Labem, Pasteurova 3632/15, Ústí nad Labem, 400 96 Czech Republic; 6 Laboratory of Molecular Biology of Bacterial Pathogens, Institute of Microbiology of the Czech Academy of Sciences, Vídeňská 1083, Prague, 14220 Czech Republic; 7 Laboratory of Structural Bioinformatics of Proteins, Institute of Biotechnology of the Czech Academy of Sciences, BIOCEV Research Center, Prumyslova 595, Vestec, 252 50 Czech Republic; 8 Department of Clinical and Molecular Pathology, Faculty of Medicine and Dentistry, Palacky University Olomouc, Hněvotínská 3, Olomouc, 779 00 Czech Republic

**Keywords:** IL-6, IL-6R blockers, Cancer cell migration, Migrastatics, Malignant melanoma, Pancreatic carcinoma, GAMG glioblastoma, HEK-Blue IL-6, Protein engineering

## Abstract

**Background:**

Interleukin-6 (IL-6) is a multifunctional cytokine that controls the immune response, and its role has been described in the development of autoimmune diseases. Signaling via its cognate IL-6 receptor (IL-6R) complex is critical in tumor progression and, therefore, IL-6R represents an important therapeutic target.

**Methods:**

An albumin-binding domain-derived highly complex combinatorial library was used to select IL-6R alpha (IL-6Rα)-targeted small protein binders using ribosome display. Large-scale screening of bacterial lysates of individual clones was performed using ELISA, and their IL-6Rα blocking potential was verified by competition ELISA. The binding of proteins to cells was monitored by flow cytometry and confocal microscopy on HEK293T-transfected cells, and inhibition of signaling function was examined using HEK-Blue IL-6 reporter cells. Protein binding kinetics to living cells was measured by LigandTracer, cell proliferation and toxicity by iCELLigence and Incucyte, cell migration by the scratch wound healing assay, and prediction of binding poses using molecular modeling by docking.

**Results:**

We demonstrated a collection of protein variants called NEF ligands, selected from an albumin-binding domain scaffold-derived combinatorial library, and showed their binding specificity to human IL-6Rα and antagonistic effect in HEK-Blue IL-6 reporter cells. The three most promising NEF108, NEF163, and NEF172 variants inhibited cell proliferation of malignant melanoma (G361 and A2058) and pancreatic (PaTu and MiaPaCa) cancer cells, and suppressed migration of malignant melanoma (A2058), pancreatic carcinoma (PaTu), and glioblastoma (GAMG) cells in vitro. The NEF binders also recognized maturation-induced IL-6Rα expression and interfered with IL-6-induced differentiation in primary human B cells.

**Conclusion:**

We report on the generation of small protein blockers of human IL-6Rα using directed evolution. NEF proteins represent a promising class of non-toxic anti-tumor agents with migrastatic potential.

**Supplementary Information:**

The online version contains supplementary material available at 10.1186/s12964-024-01630-w.

## Background

Interleukin-6 (IL-6) is a pleiotropic cytokine that orchestrates multiple physiological processes. IL-6 is produced by many cells in the human body, and it is recognized by two types of cognate receptors: transmembrane and soluble [1]. IL-6 signaling is important to mediate immune responses; however, it could be misused in autoimmune diseases [2], cancer progression [3], and serious infections [4], where it can cause cytokine storm and organ failure, as observed in the COVID-19 pandemic. Thus, the IL-6 cytokine and its cognate receptor have attracted attention as important therapeutic targets [2, 4].

The IL-6 receptor complex comprises two subunits, namely interleukin-6 receptor α (IL-6Rα) and glycoprotein 130 (gp130). IL-6Rα is a non-signaling subunit that exclusively binds to IL-6. On the other hand, gp130 is a signal-transducing subunit that is shared among IL-6 family cytokines. Signaling receptor complex assembly occurs in three steps. The initial step involves the binding of IL-6 to the IL-6Rα subunit, followed by IL-6/IL-6Rα assembly with gp130. Finally, two IL-6/IL-6Rα/gp130 trimers form a hexameric complex that ensures gp130 dimerization and signal transduction [5, 6]. While gp130 is abundant in most cells of the body, membrane IL-6Rα expression is restricted to a few cell types [7]. However, IL-6 can also initiate signaling with soluble IL-6Rα, thus broadening the responsive cell type repertoire [8]. IL-6 activates several downstream pathways, but mainly Janus kinase/signal transducer and activator of transcription (JAK/STAT) [9]. Other pathways utilized for IL-6 signaling are mitogen-activated protein kinase (MAPK), phosphoinositide 3-kinase (PI3K)/Akt, and gp130/SFK/YAP [10, 11].

Multistage involvement of IL-6 in complex physiological processes has a downside. Normally, IL-6 signaling should fade out after stress resolution. However, dysregulated IL-6 signaling causes chronic inflammation and disturbs tissue homeostasis, leading to tissue damage and loss of function [12, 13]. Similarly, IL-6 also contributes to cancer development [3, 9, 14]. The IL-6 effects, which are beneficial during wound healing, are turned against the organism during tumorigenesis; hence, processes that occur during tumor development resemble those in wound healing. In both cases, IL-6 promotes cell proliferation, tissue remodeling, cell migration, and angiogenesis. Furthermore, IL-6 attracts the immune-suppressive M2 macrophages and stimulates fibroblast differentiation into cancer-associated fibroblasts (CAFs) with a myofibroblast phenotype, thus shaping tumor microenvironment (TME) [15]. The accumulation of knowledge about the role of IL-6 in normal and pathological conditions led to the hypothesis that IL-6 blocking could be a viable therapeutic strategy for some diseases. Initially, this therapeutic option was investigated in the context of autoimmunity. The first IL-6 inhibitor, Tocillizumab (TCZ; RoActemra® or Actemra®), which blocks IL-6Rα, was approved by the Food and Drug Administration (FDA) for rheumatoid arthritis treatment [16]. However, the applicability of IL-6 inhibition to other diseases, including cancer, was also investigated. The inhibition of IL-6 can affect cancer cell proliferation and metastasis by influencing cancer cells directly or via TME. Alternatively, IL-6 antagonists can be used in combination with other medicines [17].

The IL-6Rα inhibitors are mostly represented by monoclonal antibodies (mAbs) such as TCZ, Sarilumab, and Satralizumab. Several other mAbs and small molecules are in pre-clinical and clinical trials [18]. However, single-domain protein scaffolds are valuable alternatives that offer significant benefits such as fast tissue penetration and easy molecular modification. Recently, we developed a collection of small protein blockers derived from a three-helix scaffold of the albumin-binding domain (ABD) of streptococcal protein G [19] that were targeted to human IL-23 receptor and IL-17 receptor A [20, 21]. Herein, we describe the development of a set of ABD-based IL-6Rα binding proteins that exhibited a blocking effect on IL-6-mediated signaling in vitro. Our data further underscore the role of IL-6 in cancer cell proliferation and migration, and therefore can be used as a molecular clue for the development of more efficient anti-cancer therapeutics.

## Materials and methods

### Antibodies, recombinant proteins, and detection agents

Human (h) IL-6Rα, anti-mouse mAb-horseradish peroxidase (HRP), neutralizing anti-hIL-6R1 mAb, anti-mouse HRP-conjugated antibody, mouse IgG1κ isotype (anti-hIL-23 (p19)) were obtained from BioLegend, San Diego, CA, USA. hIL-6, anti-hIL-6R1 rabbit polyclonal antibody (pAb), and anti-Avi-Tag mouse mAb were obtained from antibodies-online, Aachen, Germany. Anti-hIL-6 rabbit pAb was obtained from AssayPro, St. Charles, AR, USA. Streptavidin-phycoerythrin (PE) was purchased from eBioscience, San Diego, CA, USA. Isotype control antibody MOPC-21 (mouse IgG1) was obtained from EXBIO Praha, a.s., Vestec, Czech Republic. Alexa Fluor 647-conjugated goat anti-mouse IgG F(ab’)2 fragment (GAM-AF647) was purchased from Jackson ImmunoResearch Laboratories, West Grove, PA, USA. Pierce High Sensitivity Streptavidin-HRP and anti-CD19 mAb PE-Alexa Fluor 610 were purchased from Thermo Fisher Scientific, Waltham, MA, USA. Anti-CD38 mAb PE/Dazzle 594 was obtained from PerkinElmer, Waltham, MA, USA. PE anti-CD126 (IL-6Rα) mAb was obtained from Sony Biotechnology, San Jose, CA, USA. Anti-pStat3 (Tyr705) rabbit mAb and anti-rabbit HRP-conjugated antibody were purchased from Abcam, Cambridge, United Kingdom. Anti-Stat3 mouse mAb was obtained from Cell Signaling Technology, Danvers, MA, USA. Anti-α-Tubulin mouse antibody was purchased from Sigma-Aldrich, St. Louis, MO, USA. Goat anti-rabbit Abberior STAR RED was purchased from Abberior, Göttingen, Germany.

### HEK-Blue cell line and growth conditions

HEK-Blue IL-6 reporter cell line (InvivoGen, San Diego, CA, USA) used in the study was cultured in Dulbecco’s modified Eagle’s medium (DMEM) (BioSera, Cholet, France) containing 2 mM L-glutamine and 4.5 g/l glucose, supplemented with 10% heat inactivated fetal bovine serum (FBS), and antibiotics (100 U/ml penicillin, 100 µg/ml streptomycin, 100 µg/ml Normocin (InvivoGen, San Diego, CA, USA), and HEK-Blue Selection (InvivoGen, San Diego, CA, USA)) at 37 °C in 5% CO_2_. For the fluorescent microscopy, medium without Normocin and HEK-Blue Selection was used. For the signaling inhibition experiments, DMEM with 100 U/ml penicillin and 100 µg/ml streptomycin was used.

### Ribosome display selection

According to the ABD-derived scaffold design, 11 residues of the ABD wild type (ABDwt) domain were randomized. The combinatorial NNK library was generated by assembly PCR and purified on 1% agarose gel, as described previously [22]. The gene construct, which was used for the ribosome display selection, contained the T7p (Bacteriophage T7 RNA Polymerase Promoter), ribosome binding site (RBS), ABD variant, TolA spacer, and lacks stop codon. The ribosome display protocol was adapted from Pluckthun’s laboratory protocol [22]. Ribosome display selection was carried out on the MaxiSorp immune plate (Nunc A/S, Roskilde, Sjælland, Denmark). hIL-6Rα at a concentration of 25 µg/ml was coated overnight in carbonate buffer (35 mM Na_2_CO_3_, 14 mM NaHCO_3_, pH 9.6), washed with TBS buffer (50 mM Tris-HCl, 150 mM NaCl, pH 7.4), and blocked with 3% BSA in TBS for 1 h at room temperature (RT). Following assembly, the combinatorial library was transcribed and translated using the PURExpress In Vitro Protein Synthesis Kit (NEB, Ipswich, MA, USA) according to the manufacturer’s instructions. 1 µg of DNA was used per 50 µl reaction. WBT buffer (50 mM Tris-acetate, 150 mM NaCl, 50 mM MgAc, pH 7.0) with 0.5% BSA and 2.5 mg/ml heparin was added to the translation mixture. Additionally, ABDwt was added to the library mixture as a blocking agent to prevent unspecific binding of the ABD variants to the highly heterogeneous MaxiSorp surface. Then, library was transferred to a well coated with 3% BSA for pre-selection at 4 °C for 1 h. For selection, the library was transferred to a well coated with hIL-6Rα for 1 h at 4 °C. Unbound variants were washed with WBT buffer containing Tween20. A varied number of wash cycles and Tween20 concentrations were used in each selection round (Table S1). mRNA of the selected variants was released from ribosomes by elution buffer (50 mM Tris-acetate, 150 mM NaCl, 50 mM EDTA, pH 7.5), containing 50 µg/ml *S. cerevisiae* RNA and 2.5 mg/ml heparin. mRNA was purified using the High Pure RNA Isolation Kit (Roche, Basel, Switzerland) and transcribed to cDNA using GoScript Reverse Transcriptase (Promega, Madison, WI, USA). The library was assembled for the next round of selection using the same PCR assembly steps as in Ref [22], and the next round of selection followed. Three rounds were carried out. Finally, the enriched ABD library was cloned into the pET28 vector using NcoI (NEB, Ipswich, MA, USA) and BamHI HF (NEB, Ipswich, MA, USA) restriction endonucleases. The obtained plasmid library was called NEF, and gene constructs included HisTag – NEF variant – FlagTag – TolA – AviTag. Finally, the plasmids were introduced into the *E. coli* BL21-Gold (DE3) strain for protein production, and individual bacterial clones were used for ELISA screening.

### Protein production

The overnight cultures of individual bacterial colonies of *E. coli* BL21-Gold (DE3) transformed with a gene of interest were grown in Luria-Bertani (LB) media with 60 µg/ml of kanamycin (Km) overnight at 37 °C. Overnight culture was inoculated in LB medium with Km at 50 times dilution. After the bacterial culture had reached OD_600_ = 0.6, protein expression was induced with 1 mM isopropyl β-d-1-thiogalactopyranoside (IPTG) for 4 h at 37 °C. Bacterial cultures were centrifuged at 7000×g and 4 °C for 10 min. The cell pellets were harvested and stored at -20 °C. For the production of biotinylated protein in the *E. coli* BirA strain, a slightly modified protocol was used. Overnight cultures were grown in LB containing both Km and 30 µg/ml of chloramphenicol (Chp). Then, 50 mM d-biotin was added for 10 min. Protein production was induced with 1.5 mM IPTG for the next 4 h at 37 °C.

### Bacterial lysate preparation

The harvested cell pellets were resuspended in lysis buffer (50 mM Tris-acetate, 300 mM NaCl, pH 7.4). Then, cells were disrupted on an ice bath by sonication using Misonix S3000 sonicator with the following program: total ON time 1-5 min; 5s ON/10s OFF; Power 12 W. Following, the cell lysate was centrifuged at 18,000×g and 4 °C for 20 min to remove cell debris.

### Protein purification

Proteins were purified from a lysate using affinity chromatography with Ni-NTA agarose (Qiagen, Hilden, Germany) according to the manufacturer’s protocol. Briefly, cell lysate supernatant collected in the previous step was applied to 1 ml of Ni-NTA agarose, and flow-through was collected. The procedure was repeated three times, and the protein captured on Ni-NTA agarose was washed with the wash buffer (50 mM Tris-acetate, 300 mM NaCl, 20 mM imidazole, pH 8.0). Protein was eluted from Ni-NTA agarose with 1 ml of elution buffer (50 mM Tris-acetate, 150 mM NaCl, 250 mM imidazole, pH 8.0) per fraction and stored at 4 °C. To reduce endotoxin concentration, an additional purification step with isopropanol (50 mM Tris-acetate, 60% isopropanol, pH 8.0) was applied. To prepare endotoxin-free protein isolate, Polymyxin B-Agarose (Sigma-Aldrich, St. Louis, MO, USA) was used after Ni-NTA purification with isopropanol.

### ELISA screening

The NEF proteins were produced in 5 ml *E. coli* BL21-Gold (DE3) bacterial culture and 1 ml cell lysates were used for the ELISA screening. Briefly, hIL-6Rα (2 µg/ml in carbonate buffer) was immobilized on the MaxiSorp plate at 4 °C overnight and blocked with Pierce Protein-Free Blocking Buffer (Thermo Fisher Scientific, Waltham, MA, USA) for 1 h at RT. Under similar conditions, lysozyme (2 µg/ml) was immobilized to test the specificity of the NEF variants. The bacterial lysate containing the NEF variant was 4,000 times diluted in PBSTB (PBS amended with 0.05% Tween20 and 1% BSA) and added in the following ELISA step. Next, after thrice washing with PBST (PBS with 0.05% Tween20), the NEF variant detection was carried out using α-Avi-Tag mouse mAb (1:5,000) and α-mouse mAb-HRP (1:5,000) in PBSTB. Following, 3,3’,5,5’-Tetramethylbenzidine (TMB) (TestLine, Brno, Czech Republic) substrate was added and incubated for 30 min in the dark at RT. Then, the reaction was stopped using 2 M H_2_SO_4_ and absorbance at 450 nm wavelength was measured for the degraded substrate using Epoch 2 microplate spectrophotometer (BioTek, Santa Clara, CA, USA).

### Sequence analysis of selected variants

Plasmids containing NEF variants were isolated using the QIAprep Spin Miniprep Kit (Qiagen, Hilden, Germany). DNA was eluted from a column using sterile water. Plasmids were sequenced using the pETup primer (5’-ATGCGTCCGGCGTAGA-3’). Sequencing data were analyzed using SnapGene software (GSL Biotech LLC, San Diego, CA, USA).

### Binding ELISA

For binding ELISA, NEF variants were expressed in 100 ml culture of the *E. coli* BirA strain, and proteins were extracted and purified from lysates, as mentioned in earlier sections. Briefly, ELISA was carried out using hIL-6Rα (5 µg/ml in carbonate buffer) immobilized on the PolySorp plate overnight at 4 °C (Nunc A/S, Roskilde, Sjælland, Denmark). Also, BSA was immobilized to test the specificity of the NEF variants. Following, plates were blocked with PBSTB for 2 h at RT. Then, serially diluted, 5 times per step, NEF variants were added to hIL-6Rα and further incubated for the next 1 h RT. Neutralizing anti-hIL-6R1 mAb was used as a positive control, while mouse IgG1κ isotype (anti-hIL-23 (p19)) and ABDwt were used as negative controls. Detection was carried out using Pierce High Sensitivity Streptavidin-HRP (1:10,000) and anti-mouse mAb-HRP (1:5,000) in PBSTB. TMB was added and signal was detected as reported in previous section.

### Competition ELISA

To perform competition ELISA, hIL-6Rα (1.5 µg/ml in carbonate buffer) was immobilized on the MaxiSorp plate overnight at 4 °C. Then, the plate was blocked with Pierce Protein-Free Blocking Buffer for 1 h at RT. Next, 0.25 µg/ml of hIL-6 together with an increasing concentration of the purified NEF variants in PBSTB was added and incubated for the next 1 h at RT. Anti-hIL-6R1 mAb was used as a positive control, while mouse IgG1κ isotype (anti-hIL-23 (p19)) and ABDwt were used as negative controls. Finally, hIL-6 was detected using anti-hIL-6 rabbit pAb (1:1,000) and anti-rabbit pAb-HRP conjugate (1:4,000) in PBSTB. TMB was added and signal was detected as reported in previous section.

### Confocal microscopy

HEK-Blue IL-6 reporter cells were seeded on the sterile 24-well plate (TPP, Trasadingen, Switzerland) and cultured overnight. Following, hIL-6 (10 ng/ml concentration) was added to the cell culture and incubated for the next 3 h, adapted from Ref [23]. Meanwhile, in vivo biotinylated NEF variants (10 µg/ml or 250 nM concentration) were mixed with 4 µg/ml of Streptavidin-conjugated Alexa Fluor 568 (Invitrogen, Waltham, MA, USA) in DMEM, incubated for 30 min at RT, and centrifuged at 18,000×g for 10 min at RT. Afterwards, the NEF/Streptavidin complex was added to HEK-Blue IL-6 reporter cells and incubated for 5 h at 37 °C. Then, cells were washed five times with PBS and fixed with 4% paraformaldehyde (PFA) for 15 min at RT. Imaging was performed using the Zeiss LSM 780 confocal microscope. Under similar conditions, the HEK293T cell line was treated and used as a negative control to investigate the NEF specificity.

Additionally, NEF proteins detection on the hIL-6Rα-transfected HEK293T cells was also performed. Briefly, HEK293T cells were seeded on 18-mm cover glass (P-Lab, Prague, Czech Republic). After reaching 80% confluence, cells were transfected with the *hIL-6R*α gene in the pcDNA6 vector, 1 µg DNA per transfection. Plasmid DNA was mixed with PEI at a ratio of 1:4 (w/w) and incubated for 20 min at RT. This mixture was then added to HEK293T cells in a serum-free medium, followed by 6 h incubation at 37 °C. Afterwards, the medium was exchanged with complete DMEM medium, and cells were incubated for the next 48 h. HEK293T cells treated with PEI reagent alone (no DNA) were used as a mock control. Meanwhile, the NEF variants were labeled with fluorescein isothiocyanate (FITC) (Sigma-Aldrich, St. Louis, MO, USA), where 50 ng of FITC in dimethyl sulfoxide (DMSO) was used per 1 µg of protein, and labeling was performed in carbonate buffer (pH 9.6) for 90 min at 37 °C. HEK293T (48 h post-transfection) cells were incubated with 40 µg/ml (1 µM) FITC-labeled NEF variants in DMEM for 1 h RT. Next, cells were washed five times with PBS and fixed with 4% PFA for 15 min. Afterwards, cells were washed three times with PBS and blocked with 1.5% BSA in PBS for 30 min. Following, cells were incubated with 5 µg/ml anti-hIL-6R1 rabbit pAb and 1 µg/ml goat anti-rabbit Abberior STAR RED antibody in PBST with 1.5% BSA. Finally, cells were transferred to the glass slide (P-Lab, Prague, Czech Republic) with mounting medium Vectashield with DAPI (Vector Laboratories, Newark, CA, USA). Imaging was performed using the Carl Zeiss LSM 880 NLO confocal microscope.

### HEK-Blue IL-6 reporter cell assay

For the HEK-Blue IL-6 reporter assay, 3.6 × 10^4^ HEK-Blue IL-6 cells in 180 µl volume per well were seeded on a sterile 96-well cell culture plate (Nunc A/S, Roskilde, Sjælland, Denmark). Then, cells in each well were incubated with 2–5 ng/ml hIL-6 for 21 h in the presence of an increasing concentration (up to 10 µM) of immobilized metal affinity chromatography (IMAC)-purified NEF protein or neutralizing antibody (TCZ or anti-hIL-6R1 mAb) in 20 µl volume. After the incubation, 20 µl of cell supernatant was mixed with 180 µl of the Quanti-Blue Solution and incubated for 3 h at 37 °C in the dark. To detect the secreted SEAP, absorbance at 620 nm was measured with Epoch 2 microplate spectrophotometer.

### Flow cytometry assay

Cultured HEK-Blue IL-6 and HEK293T cells were collected and washed in HEPES-buffered salt solution (HBSS buffer; 10 mM HEPES, 140 mM NaCl, 5 mM KCl, pH 7.4) supplemented with 2 mM CaCl_2_, 2 mM MgCl_2_, 1% (w/v) glucose, and 1% (v/v) FCS (cHBSS buffer). 2 × 10^5^ cells/sample in HBSS-Ca/Mg buffer was incubated with 10 µg/ml of biotin-labeled ligands (NEFs and ABDwt) for 30 min at 4 °C. Following this, the cells were washed with cHBSS buffer and then incubated with PE-labeled Streptavidin (1:400) at 4 °C for 30 min. Next, cells were washed and resuspended in cHBSS buffer, and finally investigated by flow cytometry using a FACS LSR II instrument (BD Biosciences, San Jose, CA, USA) in the presence of 1 µg/ml of Hoechst 33258. Then, the collected data was processed with appropriate gatings to exclude debris, cell aggregates, and dead cells (Hoechst 33258-positive staining) using the FlowJo software (BD Biosciences, Franklin Lakes, NJ, USA). The binding data was deduced from the mean fluorescence intensities (MFI) of cell-bound ligands and expressed as relative values, with the highest MFI value of the ligand taken as 100%. For antibody binding, 2 × 10^5^ cells/sample were incubated with anti-hIL-6R1 mAb (1:100) or IgG1κ isotype control (1:100) in HBSS-Ca/Mg buffer at 4 °C for 30 min. Next, cells were washed and incubated with the GAM-AF647 antibody (1:500) at 4 °C for 30 min. After that, cells were washed and resuspended in cHBSS buffer and then analyzed by flow cytometry as described above.

### LigandTracer assay

One day before the transfection, 1 × 10^6^ HEK293T cells were seeded on a Petri dish (Nunc A/S, Roskilde, Sjælland, Denmark) and incubated overnight in a slant position in 5% CO_2_ incubator. Transfection with *hIL-6Rα* was carried out as above. Finally, after 18 h of post-transfection, binding of the NEF binders to the cell surface expressed hIL-6Rα was measured using the LigandTracer Green Line instrument (Ridgeview Instruments AB, Uppsala, Sweden) coupled with a Red (632 nm) - Near-infrared (NIR; 670 nm) detector. Herein, detection of the fluorescence signal corresponding to the in vivo biotinylated NEF binders was done as follows: (i) the baseline measurement was performed in the absence of the NEF proteins and fluorophores (only DMEM medium) for at least 15 min; (ii) during association phase, the fluorescence signal after addition of the in vivo biotinylated NEF variants preincubated with Streptavidin-APC conjugate was measured for at least 30 min (until the signal reached saturation state); and (iii) during dissociation phase, the measurement of the signal after medium exchange (DMEM only) was performed for at least 30 min (until the signal intensity was significantly reduced). Finally, the binding kinetics and ‘One-to-one’ or ‘One-to-one depletion corrected’ evaluation methods were applied for the calculation of kinetic parameters (ka, kd, and K_D_) using TraceDrawer 1.7.1 software.

For competition assay, NEF variants binding to the *hIL-6Rα-*transfected HEK293T cells was detected in absence or presence of hIL6 or TCZ using above protocol with slight modifications. Briefly, upon stabilization of baseline fluorescence signal, association phase was initiated by addition of selected concentration of in vivo biotinylated NEF variant to the cells. During the association phase, an increasing concentration of hIL6 (25 and 100 nM) or TCZ (3, 50 and 300 nM) were added to the cells at a specific interval (ca. every 30 min). Finally, the dissociation was performed by exchanging the culture medium containing NEF variants, hIL6 or TCZ with the fresh medium without additives. Under similar conditions, non-transfected HEK293T cells were treated with NEF variants and used as a negative control to investigate the NEF specificity.

### Binding of NEF proteins to primary B cells

Peripheral blood mononuclear cells (PBMCs) isolation was performed in Ficoll-Paque PLUS medium (VWR, Radnor, PE, USA) using density gradient centrifugation. To get activated B cells, 2 × 10^6^ PBMCs/ml were cultured in DMEM supplemented with 10% FBS, penicillin/streptomycin, and 5 µg PWM/ml at 37 °C for 96 h in 5% CO_2_ incubator. Unstimulated PBMCs were grown in the same cultivation medium without PWM under similar culture conditions. Next, cells were divided into aliquots – 2 × 10^5^ cells per aliquot. Each aliquot was stained with anti-CD19 mAb PE-Alexa Fluor 610, anti-CD38 mAb PE/Dazzle 594, and 1 µg of NEF binder or 5 µl of PE anti-CD126 (IL-6Rα) mAb, incubated overnight at 4 °C. For NEF binder’s detection, Streptavidin-PE conjugate antibody was incubated for 30 min at RT. Samples were measured by the Sony SP6800 Spectral Cell Analyzer (Sony Biotechnology, San Jose, CA, USA) and the data was processed using FlowJo V10 software.

### IL-6-mediated B cells differentiation inhibition assay

Herein, PBMCs were isolated as described in the NEF binding assay. Briefly, PBMCs were resuspended in complete RPMI 1640 medium containing 10% FBS and antibiotics (penicillin and streptomycin). Then, 1 × 10^6^ cells per well were mounted on the 96-well panel. First, cells were incubated with 100 nM NEF binders for 2 h, and then 10 ng/ml hIL-6 was added to the cells. Following, cells were incubated for the next 7 days. On 4^th^ day, one-half of the medium was replaced with fresh medium containing hIL-6 and NEF binders. On day 7, cells were stained for flow cytometry analysis. Herein, Fc receptors on the surface of B cells were blocked by 10% heat-inactivated human sera for 10 min at RT. After washing with PBS, anti-CD19 mAb FITC, anti-CD38 mAb PE-Texas Red, and anti-IgA mAb Pacific Blue were added to the cells, followed by 30 min incubation in the dark at RT. Finally, cells were washed with PBS and examined by the SONY flow cytometer SH800. Data was analyzed in FlowJo V10 software, and analyzed for statistical validation in GraphPad Prism software.

### Cell proliferation assay

Briefly, 5 × 10^3^ glioblastoma GAMG cells were seeded on a 96-well cultivation plate (TPP, Trasadingen, Switzerland) in 100 µl of complete DMEM. The next day, the culture medium was supplemented with a serial dilution of 1.6 µM to 0.003 µM for both the NEF binders (test) and ABDwt (negative control). Following, the cells were cultivated for the next 24 h, and the cell counting kit (CCK-8) (Sigma-Aldrich, St. Louis, MO, USA) reagent was added, followed by incubation for the next 2 h. Then, absorbance of the metabolized CCK-8 reagent was measured at 450 nm using a spectrophotometer. Finally, the proliferation rate of the cells was calculated from the calibration curve of non-treated cells, which were plated between 5 × 10^4^ to 5 × 10^2^ cells in 100 µl per well. The experiments for all the proteins were repeated twice in triplicates.

### Scratch migration assay

The polydimethylsiloxane inserts (kindly provided by University of J. E. Purkyně in Ústí nad Labem, Ústí nad Labem, Czech Republic) were placed into wells of 6-well cultivation plates. Inserts allowed the seeding and culturing of GAMG cells into two separate chambers with a 1 mm thick partition between them. Briefly, 1 × 10^5^ GAMG cells in 300 µl of complete DMEM (supplemented with 10% FBS and penicillin/streptomycin) were seeded in each chamber of the wells and incubated for the next 24 h. Then, the inserts were removed, which resulted in scratch (gap) formation in the wells. Following, the wells were rinsed twice with the culture medium to remove unattached cells, and the cell growth in the scratch area was checked using light microscopy. Next, the culture medium was replaced with a mixture of 2 ml of DMEM supplemented with 200 nM NEF binders (test) and ABDwt (negative control). Also, half of the samples were incubated with hIL-6 (50 ng/ml). Following, the cells were allowed to migrate in the scratch area by incubation at 37 °C for 48 h in 5% CO_2_ incubator. Finally, all the wells were washed with fresh medium to remove unattached cells, and cell migration was visualized using an Olympus light microscope. All the captured images were evaluated for scratch width using the ImageJ software Fiji. The experiments for all the proteins were repeated twice in triplicates.

### Cell proliferation assay by Incucyte

In this assay, 5 × 10^3^ cells (melanoma A2058 and pancreatic PaTu cancer cell lines) per well in DMEM enriched by NEF binders or controls were seeded on the 96-well plates. The following day, the growth medium was replaced, and continuous screening was initiated using Incucyte S3 Live-Cell Analysis System (Sartorius Lab Instruments GmbH & Co. KG, Goettingen, Germany). All experiments were performed in six technical replicates (wells) using four defined points for confluence measurement every 2 h for the next four consecutive days. The resulting confluence was determined using the Incucyte Cell-by-Cell Analysis Software Module (Sartorius Lab Instruments GmbH & Co. KG, Goettingen, Germany), and data (in%) were exported for statistical analysis.

### Proliferation and cytotoxicity measurements by iCELLigence

Herein, 5 × 10^4^ cells (human primary fibroblasts, melanoma (G361 and A2058) and pancreatic (PaTu and MiaPaCa) cancer cell lines) per well in DMEM enriched by NEF binders or controls, were seeded on the E-plates L8 (8 wells). Next, the continuous cell screening was initiated using the Real-Time Cellular Analysis (RTCA) iCELLigence instrument (Agilent Technologies, Inc., Santa Clara, CA, USA) for four consecutive days in standard incubator conditions. All experiments were performed in two technical replicates (wells); visualization and analysis were performed using RTCA software, the proliferation/cytotoxicity protocol, and normalized for presentation as the Delta Cell Index according to manufacturer instructions.

### Migration (wound healing) assay by Incucyte

In this assay, cells (melanoma A2058 and pancreatic PaTu cancer cell lines) were seeded at 7 × 10^4^ per well in 96-well plates. The next day, the medium was replaced, and cells were preincubated with inhibitors overnight. Afterwards, standardized wounds were created using Incucyte WoundMaker - a 96-pin mechanical device, and continuous screening was initiated using the Live-Cell Analysis System Incucyte S3. All experiments were performed in six technical replicates (wells) using two defined points for wound size measurement every 2 h up to three consecutive days. The resulting wound healing data acquired using the Incucyte Scratch Wound Analysis Software Module was exported and analyzed for statistical analysis.

### Analysis of pSTAT3 activity in cancer cells

Pancreatic carcinoma (PaTu) cells were seeded at a density of 2 × 10^4^/cm^2^ in a culture medium (DMEM supplemented with 10% FBS) and incubated for 24 h to fully attach and initiate proliferation. Consequently, the medium was replaced with a new complete medium enriched with NEF variants or ABDwt control, respectively. After overnight preincubation, cells were stimulated with hIL-6 (10 ng/ml) for 15 min and analyzed immunocytochemically. The phosphorylated STAT3 (pSTAT3) cell staining results were scored visually based on weighted intensity (assuming 0 for no staining, 1 for weak staining, 2 for moderate staining, and 3 for strong staining).. Mitotic cells are highlighted by black arrows. Examples of weakly positive nuclei (intensity = 1) are indicated by empty arrowheads, and medium-to-strongly positive nuclei (intensity = 2–3) are indicated by full black arrowheads.

### Western blot

In this study, 6 × 10^5^/ml U87MG cells in 2 ml were seeded on the 6-well plate overnight. Following, cell medium was exchanged with FCS-free DMEM and further incubated for 9 h at 37 °C. Then, pSTAT3 was induced by 100 ng/ml of hIL-6 in the presence of increasing concentrations of NEF variants, ABDwt, or TCZ in serum-free medium for 15 min. Next, cells were washed with ice-cold PBS and harvested with 100 µl of lysis buffer (25 mM Tris, 150 mM NaCl, 1 mM EDTA, 1% Triton, 4 mM Na_3_VO_4_, pH 7.4) supplemented with protease inhibitor cocktail (1:100) (Sigma-Aldrich, St. Louis, MO, USA). After that, cell lysis was carried out on ice for 30 min and samples were centrifuged using 18,000×g at 4 °C for 10 min. The supernatant was used for protein quantification with the BCA assay (Thermo Scientific, Waltham, MA, USA). Afterwards, protein (45 µg of total protein per well) was mixed with sample loading buffer (200 mM Tris-HCl, 20% Glycerol, 10% SDS, 0.05% bromophenol blue, 125 mM DTT, pH 6.8) and heated for 5 min at 95 °C. Subsequently, proteins were separated using 12% SDS-PAGE gel electrophoresis. Then, gel was transferred onto a nitrocellulose membrane (0.2 μm, Bio-Rad, Prague, Czech Republic) and blocked with 5% milk PBST (0.1% Tween20). Anti-pStat3 (Tyr705) rabbit mAb (1:2,000) and anti-rabbit HRP-conjugated antibody (1:2,000) were then used to distinguish pSTAT3. Following, the membrane was incubated with SuperSignal West Pico PLUS Chemiluminescent Substrate (Sigma-Aldrich, St. Louis, MO, USA) at RT for 1.5 min to detect the specific pSTAT3 bands. Then, imaging was made with Azure 280 (Azure Biosystems, Sierra Court Suites, AB, USA). Antibodies were stripped using stripping buffer (12 mM glycine, 50 mM NaCl, pH 2.8) for 30 min at RT. After repeated blocking with 5% milk PBST, a total STAT3 was also detected with anti-Stat3 mouse mAb (1:1,000), and Tubulin was detected with anti-α-Tubulin mouse antibody (1:100). Finally, the anti-mouse HRP-conjugated antibody (1:2,000) was used for detection as described above.

### Molecular modeling

We modeled the structure of the ABD-derived NEF binders based on the structure of the wild type ABD (pdb id 1gjt [24]) as the template using the MODELLER 9v14 software suite [25]. The IL-6Rα structure was obtained from the crystal structure of the ternary IL-6/IL-6Rα/IL-6R beta (β) complex (pdb id 1p9m [5]). For protein-protein docking with flexible side chains, we utilized a local version of the ClusPro server [26, 27], using chains A and C from the 1p9m structure as the receptor (corresponding to IL-6Rα domains 1 to 3, residues 24 to 321, according to the UniProt [28] record P40189 and hIL-6Rα domains 2 and 3, residues 115 to 315, according to the UniProt record P08887) and the modeled NEF variants as ligands. The docking results were visualized with PyMOL version 2.6.0 (The PyMOL Molecular Graphics System, Schrödinger, LLC, New York, NY, USA).

### Determination of thermal stability

The fluorescence shift in tryptophan and tyrosine residues of the NEF variants in the temperature gradient was measured with the NanoDSF method using the Prometheus NT.48 instrument (NanoTemper Technologies GmbH, Munich, Germany). NEF samples were prepared in PBS (pH 7.4) at a concentration of 500 µg/ml and loaded into Prometheus Standard Capillaries (NanoTemper Technologies GmbH, Munich, Germany), followed by a rise in temperature from 20 to 80 °C at a rate of 1 °C/min. The excitation power was set at 70% while the tryptophan and tyrosine fluorescence emission intensities were measured. The resulting curves were plotted as a first derivative of the 350 nm/330 nm ratio as a function of temperature. Temperature melting points were estimated from the resulting curves.

### Circular dichroism spectra measurement

Far UV circular dichroism (CD) spectra of NEF variants were measured using a Chirascan Plus spectrometer (Applied Photophysics, Surrey, UK). Samples were prepared in PBS (pH 7.4) at a concentration of 200 µg/ml. Samples were loaded into a quartz cuvette with a path length of 1 cm. The measurement was done within a range of 195–260 nm, 1 nm per step, at RT. The buffer spectra were subtracted from the resulting protein sample spectra. The analysis of CD data was done with the CDNN software (Applied Photophysics Ltd, Leatherhead, UK).

### Induction and assessment of NEF108 protection in DSS-induced acute colitis

8-9 weeks old female C57Bl/6 mice (AnLab, Prague, Czech Republic) weighing between 18 and 22 g (weight before treatment) were kept under standardized conditions at a temperature of 21 -22 °C and conditions with a 12:12-h light/dark cycle and ad libitum access to food and water. We tested the effect of NEF108 protein in preventative-therapeutic regime of Dextran sulphate sodium (DSS)-induced colitis. NEF108 protein was administered by i.p. route in form of recombinant protein solution in sterile PBS once a day. Administration of NEF108 started three days before the induction of acute colitis by providing 2.5% DSS in drinking water (w/v) DSS (MW approximately 40 kDa; TdB Labs, Uppsala, Sweden) and followed for next 4 days together with DSS. At the end of experiment, the animals were euthanized by cervical dislocation in Ketamine/Xylazine anesthesia. The length of the colon was measured between the caecum and proximal rectum. The terminal third of the colon was dissected into pieces for Real Time RT-PCR (qRT-PCR) and histochemistry. Tissues for IL-1β mRNA expression was determined according to previously reported method [29]. For the elimination of DSS residues lithium chloride RNA purification was performed [30]. IL-1ß forward primer sequence was TGCCACCTTTTGACAGTGATG and reverse primer was ATGTGCTGCTGCGAGATTTG. Tissue samples for histology were fixed in 10% neutral-buffered formalin (Merck), and paraffin-embedded. Sections were stained with hematoxylin and eosin (H&E, Merck) and classified by a pathologist without prior knowledge of treatment status of individual mouse according to the classification published by Erben et al. [31], Table S4. BX43 microscope equipped with CCD camera was employed (Olympus, Tokio, Japan). Experimental protocol was approved by Ethics Committee of the Faculty of Medicine and Dentistry (Palacky University Olomouc, Czech Republic), and the Ministry of Education, Youth and Sports, Czech Republic (MSMT-10,947/2021-3).

### Statistics and reproducibility

All the experiments were performed at least two times with a minimum of technical triplicates, unless otherwise specified. GraphPad Prism version 8.0.1 (GraphPad Software Inc., San Diego, CA, USA) or OriginLab version 2023b (OriginLab Corporation, Northampton, MA, USA) was used for the statistical analysis. Error bars indicate the mean ± standard deviation (SD), unless noted otherwise. On the generated data sets, one-way ANOVA (*p* < 0.05) with Tukey’s post-hoc test was marked as statistically significant. Respective significance values are also indicated in the figure legends.

## Results

### Identification of hIL-6Rα-binding proteins by screening of ABD combinatorial library variants

Ribosome display was used to expose the ABD library to the selection pressure. Human recombinant soluble IL-6Rα was used as a molecular target for the positive selection of protein binders in 3-round ribosome display. In each selection round, the stringency of washing conditions was increased (Table S1), and after the final selection round, an enriched cDNA library was subcloned into a pET28b vector, thus forming a plasmid library called NEF. Then, *E. coli* BL21 cells were transformed with the NEF library, and bacterial clones’ lysates were tested by ELISA on a MaxiSorp plate using anti-Avi-tag mouse mAb and anti-mouse mAb-HRP conjugate. Lysozyme was used as a negative control for the detection of non-specific NEF variants. A total of 247 NEF protein variants were screened (Fig. S1). The NEF variants that demonstrated substantial binding to hIL-6Rα were selected for verification by DNA sequencing. The selection criteria for substantial binding were absorbance higher than 0.3 a.u. and a difference in measured absorbance exceeding 25% in comparison to coated lysozyme. Accordingly, plasmids carrying 40 NEF variants were isolated and sequenced. After the sequence analysis, all the mutated and redundant NEF variants were withdrawn. Consequently, a collection of 30 unique NEF variants was obtained as a result of ELISA screening.

### Binding of NEF variants to hIL-6Rα tested by ELISA

**Fig. 1:**
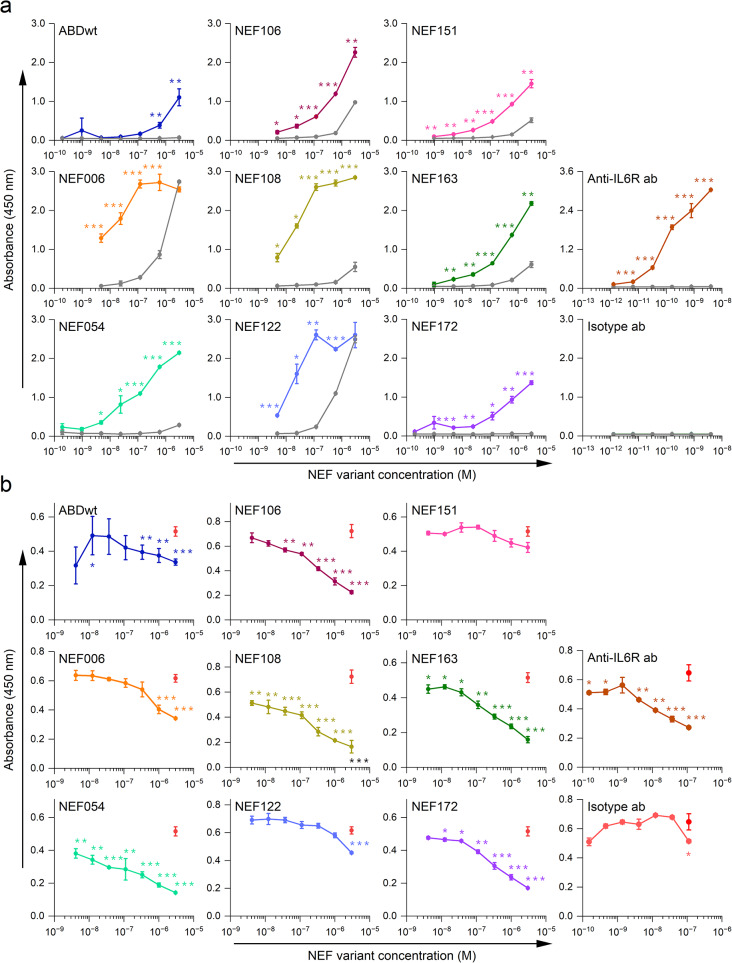
Analysis of binding specificity and affinity of selected NEF variants using ELISA. (**a**) Recombinant hIL-6Rα or BSA were immobilized on the PolySorp plate. Binding of serially diluted in vivo biotinylated NEF proteins to immobilized hIL-6Rα (colored curves) or BSA (grey curve) was detected using Streptavidin-HRP conjugate and measured at 450 nm wavelength. Binding of anti-IL6R1 mAb and isotype IgG1κ to hIL-6Rα was detected with the anti-mouse-HRP conjugate. Each point depicts the average of a duplicate with SD. Statistical significance is provided for NEF proteins binding to hIL-6Rα in comparison to BSA (**b**) For competition ELISA, hIL-6Rα was immobilized on the MaxiSorp plate, and hIL-6 cytokine with different concentrations of a purified NEF variant as sample while anti-IL6R1 mAb or irrelevant IgG1κ isotype and ABDwt as controls were also added. The hIL-6 cytokine was detected by anti-IL-6 rabbit pAb, followed by anti-rabbit IgG-HRP conjugate. Red dots represent hIL-6 binding to hIL-6Rα as a control, while each colored line represents hIL-6 binding to hIL-6Rα in the presence of serially diluted NEF protein. Each point depicts the average of the triplicate readings with respective SD. Statistical significance is provided for hIL-6 binding to hIL-6Rα in the presence or absence of the NEF variants. (**a, b**) * = *p* < 0.05; ** = *p* < 0.01; *** = *p* < 0.001; ANOVA. All experiments were performed in at least two independent experiments

The collected 30 NEF variants determined by large-scale ELISA screening were produced in *E. coli* BL21 *BirA* host cells as 38 kDa TolA fusion proteins with C-terminal biotinylation at Avi-tag. The NEF proteins were purified using IMAC chromatography and verified for specific binding to the recombinant hIL-6Rα produced in the eukaryotic expression system. The binding of serially diluted NEF variants to IL-6Rα was compared to BSA (as a negative control), which were immobilized on the PolySorp plate, and the signal was detected using Streptavidin-HRP conjugate. From the collection of 18 tested NEF variants, 12 binders exhibited a preferential binding to IL-6Rα compared to BSA (Fig. 1a). These 12 binders were selected for further analysis. The absence of ABDwt binding to hIL-6Rα indicates that binding of NEF proteins to hIL-6Rα is not an inherited property of the ABD scaffold but occurs as a result of amino acid randomization. Also, the neutralizing anti-IL6R1 mAb and mouse isotype IgG1κ binding functions were verified for the recombinant hIL-6Rα protein (Fig. 1a).

### Competition of NEF proteins with IL-6 cytokine for binding to IL-6Rα by ELISA

To identify hIL-6Rα blocking variants from the collection of selected NEF binders, a competition ELISA was performed. The soluble hIL-6Rα protein was immobilized on the MaxiSorp plate, and samples of serially diluted NEF proteins with a constant concentration of hIL-6 cytokine were added. The amount of hIL-6 bound to hIL-6Rα was then detected using anti-hIL-6 rabbit pAb followed by anti-rabbit pAb-HRP conjugate. Consequently, five NEF variants (NEF054, NEF106, NEF108, NEF163, and NEF172) (Table S2) were able to outcompete hIL-6 binding to hIL-6Rα in a concentration-dependent manner. In contrast, three other variants (NEF006, NEF122, and NEF151) and ABDwt demonstrated no inhibitory potential. The neutralizing anti-IL6R1 mAb and mouse isotype IgG1κ were used as a positive and negative controls, respectively to verify ELISA experiment design (Fig. 1b).

### Binding of NEF proteins to cell surface receptor tested by fluorescence microscopy

**Fig. 2:**
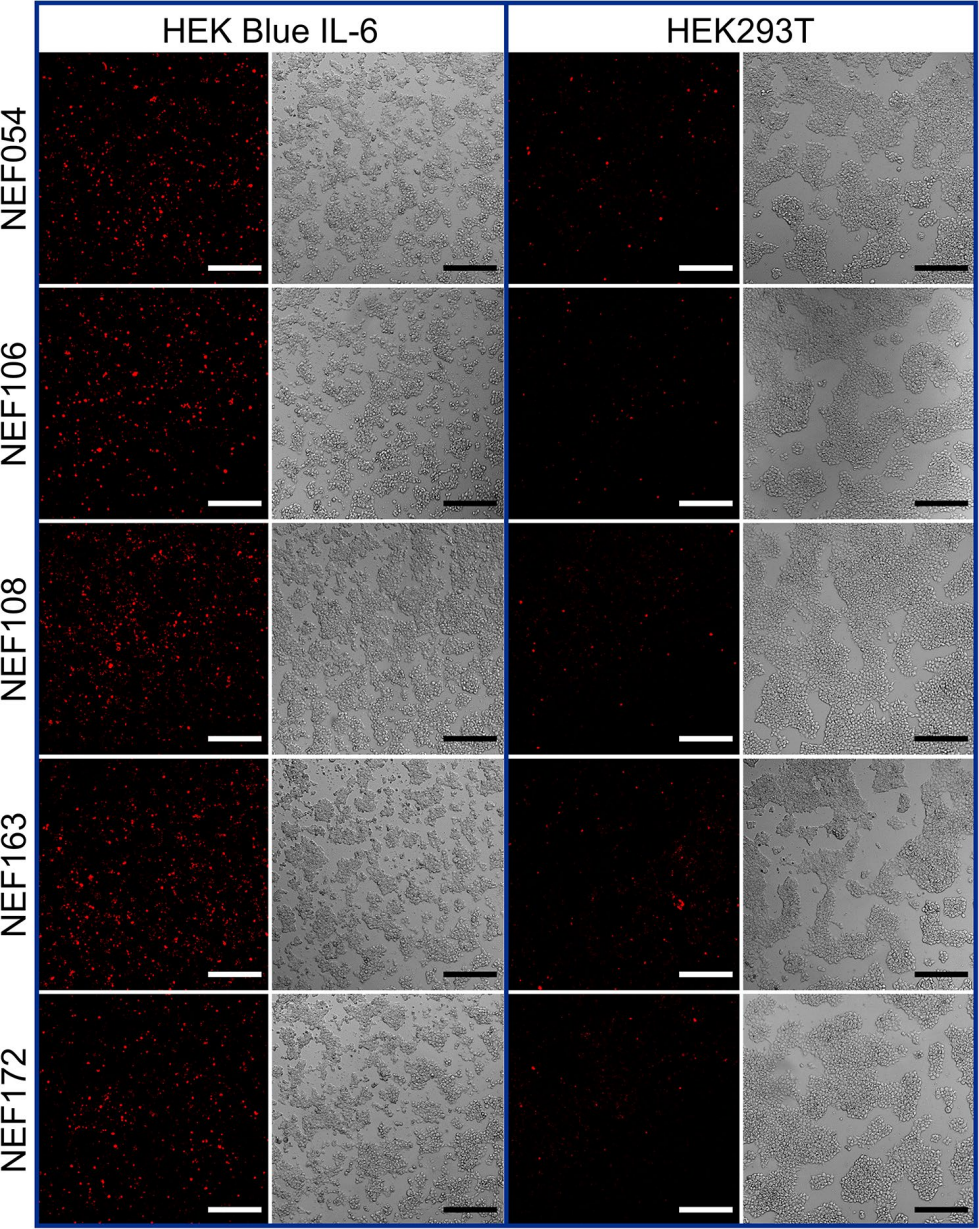
Confocal microscopy with fluorescently labeled NEF variants. In vivo biotinylated NEF proteins labeled with Streptavidin-Alexa Fluor 568 conjugate (250 nM concentration) were added to hIL-6 activated HEK-Blue IL-6 and HEK293T cells. After 5 h , cells were fixed with 4% PFA, and the binding of NEF proteins was visualized using the Zeiss LSM 780 microscope. The magnification bar represents 50 μm

To verify whether NEF proteins recognize the cell surface hIL-6Rα, HEK-Blue IL-6 cells expressing hIL-6Rα were used. The NEF054, NEF106, NEF108, NEF163, and NEF172 were produced as in vivo biotinylated products and further labeled using Streptavidin-Alexa Fluor 568 conjugate. As shown in Fig. 2 by confocal microscopy on PFA-fixed cells, all the five NEF variants exhibited substantial binding to HEK-Blue IL-6 cells compared to non-transfected HEK293T cells without hIL-6Rα expression (Fig. 2). However, hIL-6Rα expression on HEK-Blue IL-6 is rather low for detection by confocal microscopy. Thus, NEF binding to IL-6Rα on the cell surface needs to be verified by flow cytometry.

### Binding of NEF ligands to HEK-Blue IL-6 cells tested by flow cytometry

**Fig. 3:**
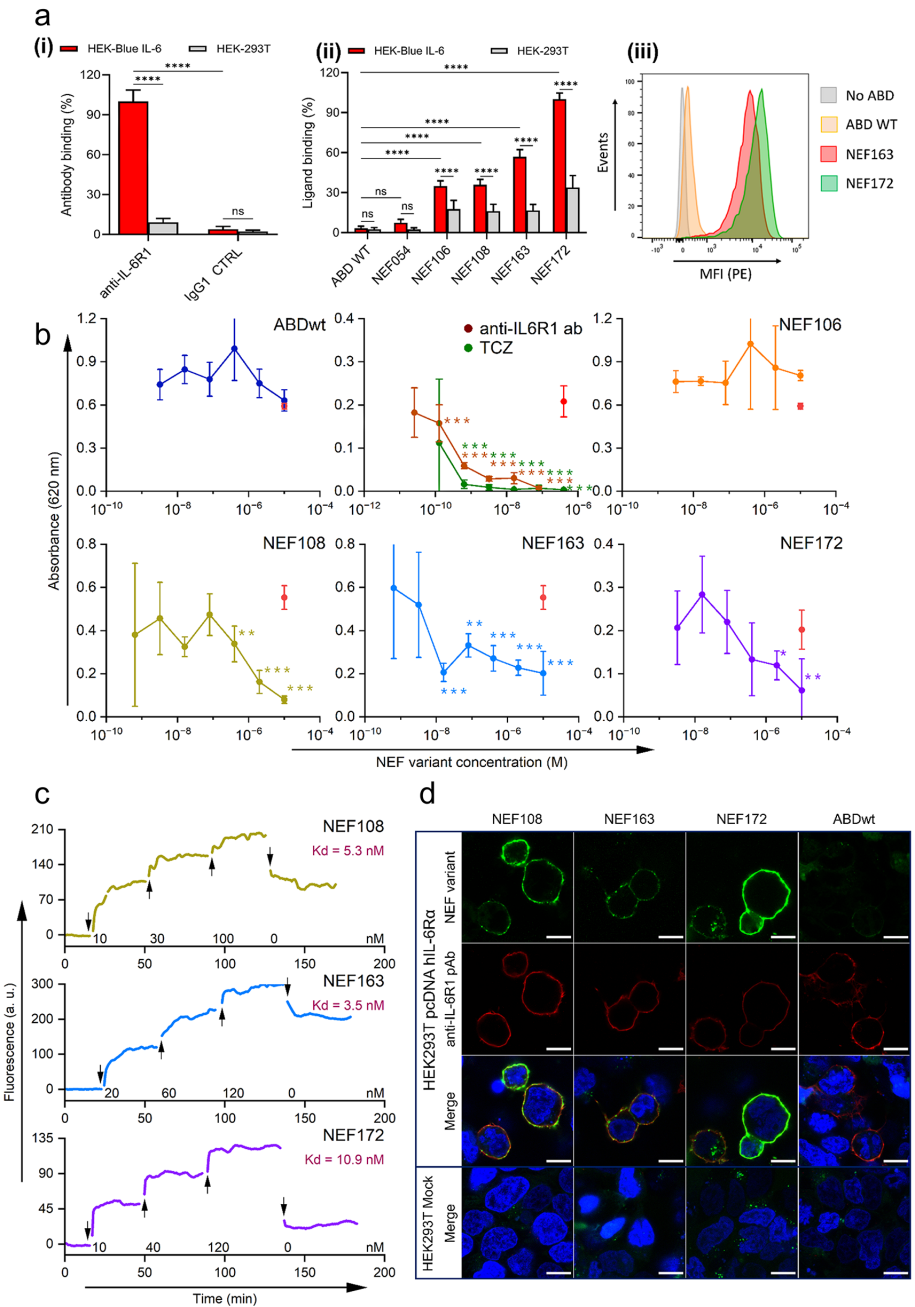
(**a**) NEF ligands bind to HEK cells expressing hIL-6Rα. (**i**) Anti-IL6R1 mAb or IgG1 isotype (IgG1 CTRL) binding to HEK-Blue IL-6 and HEK-293T. MFI for anti-IL6R1 mAb measured on HEK-Blue IL-6 was taken as 100%. (**ii**) NEF variants and ABDwt binding to HEK-Blue IL-6 and HEK293T. Data were deduced from MFI and expressed as the percentage of NEF172 binding to HEK-Blue IL-6 (taken as 100%). (**i-ii**) Bars represent the average with SD of three experiments performed in duplicate (ns, *p* > 0.05; **** = *p* < 0.0001; ANOVA). (**iii**) A typical flow cytometry histogram from a representative binding experiment to HEK-Blue IL-6 is shown. (**b**) HEK-Blue IL-6 inhibition experiment. NEF variants inhibit SEAP secretion by hIL-6-induced HEK-Blue IL-6. The absorbance of the supernatant of unstimulated cells was subtracted. ABDwt was used as a negative control, neutralizing anti-hIL-6R1 mAb and TCZ – as positive controls. The red dot represents IL-6 stimulation of HEK-Blue IL-6. Each line represents IL-6 stimulation of HEK-Blue IL-6 treated with serially diluted NEF protein. Each point depicts the average of triplicate with SD. * = *p* < 0.05; ** = *p* < 0.01; *** = *p* < 0.001; ANOVA, provides the statistical significance of IL-6R–mediated SEAP secretion in the presence of NEF or ABDwt, as well as anti-hIL-6R1 mAb and TCZ in comparison to IL-6 alone. All experiments were conducted at least twice, independently. (**c**) Kinetics and binding affinity measurements of NEF variants to cell-surface IL-6Rα using the LigandTracer. The binding of NEF variants was monitored in real-time by LigandTracer method and used to calculate K_D_. (**d**) Confocal microscopy with fluorescently labeled NEF variants. FITC-labeled (green) NEF variants’ binding was compared to ABDwt binding to *hIL-6Rα-*transfected HEK293T and Mock-transfected HEK293T. Anti-hIL-6Rα pAb (red) was used to confirm hIL-6Rα expression. The magnification bar represents 10 μm

We used HEK-Blue IL-6 cells to verify the specificity of NEF binders by flow cytometry. In Fig. 3a, HEK-Blue IL-6 cells show substantial expression of IL-6Rα (IL-6R1), as confirmed by the specific binding of anti-IL6R1 mAb, in contrast to isotype antibody control or non-transfected HEK293T cells. As further shown in Fig. 3a, NEF protein variants significantly bind to HEK-Blue IL-6 cells, except for NEF054. Of interest, the strongest binding was demonstrated by NEF172 and NEF163 proteins, while ABDwt (used as a negative control) showed no binding to HEK-Blue IL-6 cells. These results further support the specificity of the tested NEF binders to the hIL-6Rα protein.

### IL-6 inhibition cell assay

The HEK-Blue IL-6 reporter cell assay was used to investigate whether NEF variants inhibit the IL-6-mediated signaling in the cells. In the HEK-Blue IL-6 cells, hIL6-mediated signal transduction activates the JAK/STAT signaling pathway, which results in SEAP secretion in the cell culture medium that can be detected using QuantiBlue substrate. To detect inhibition of the hIL-6Rα, a constant concentration of hIL-6 was mixed with various concentrations of NEF variants and added to the reporter cells. In this assay, we investigated four NEF variants (NEF106, NEF108, NEF163, and NEF172) along with anti-hIL-6R1 mAb and TCZ (positive inhibitory controls) and ABDwt (negative control). In Fig. 3b, ABDwt did not affect hIL-6 signaling even at the highest concentration. Therefore, ABDwt does not compete with hIL-6 for hIL-6Rα binding, or induce a cytotoxic effect on HEK-Blue IL-6 cells. Likewise, no inhibitory effect was observed for NEF106. On the contrary, NEF108, NEF163, and NEF172 demonstrated a 65–70% reduction in SEAP secretion in response to hIL-6 signaling, which was relatively similar to the inhibition trend of the anti-hIL-6R1 mAb and TCZ positive controls (Fig. 3b). In particular, NEF163 showed an inhibitory effect in the concentration range of 10 nM to 10 µM, while both NEF108 and NEF172 inhibited hIL-6Rα in the range of 200 nM to 10 µM. Consequently, the competition with hIL-6 for hIL-6Rα binding observed in ELISA for NEF108, NEF163, and NEF172 was translated into functional hIL-6 signaling inhibition in the cell assay.

### Kinetics and binding affinity of NEF variants to cell surface hIL-6Rα

The *hIL-6Rα*-transfected HEK293T cells were used to monitor the NEF variants’ binding kinetics and affinity using the LigandTracer Green Line instrument. To measure association kinetics, the *hIL-6Rα*-transfected HEK293T cells were treated with several concentrations of the in vivo biotinylated NEF variants (NEF108, NEF163, and NEF172), labeled with Streptavidin-APC conjugate. After the signal saturation, the *hIL-6Rα-*transfected HEK293T cell medium containing NEF variants was replaced with a fresh medium to measure dissociation kinetics. Both NEF variant association and dissociation kinetics were then monitored in real-time by fluorescent signal detection via the Red-NIR detector (632–670 nm (ex/em)), and the resulting curves for each NEF variant were analyzed to calculate binding affinity (K_D_) (Fig. 3c). All three variants demonstrated binding affinity in a nanomolar (nM) range, where NEF163 exhibits the highest affinity (K_D_ = 3.5 nM), followed by NEF108 (K_D_ = 5.3 nM), and NEF172 (K_D_ = 10.9 nM).

Further, to verify the cell surface binding of NEF variants on *hIL-6Rα-*transfected HEK293T cells, we used confocal microscopy to visualize the fluorescently labeled NEF108, NEF163, and NEF172 variants. We found that all three NEF variants bind to cell surface hIL-6Rα, as this binding co-localizes with staining of anti-hIL-6R1 rabbit pAb (Fig. 3d).

### Inhibitory effect of NEF binders on hIL-6Rα-mediated pSTAT3 signaling

**Fig. 4:**
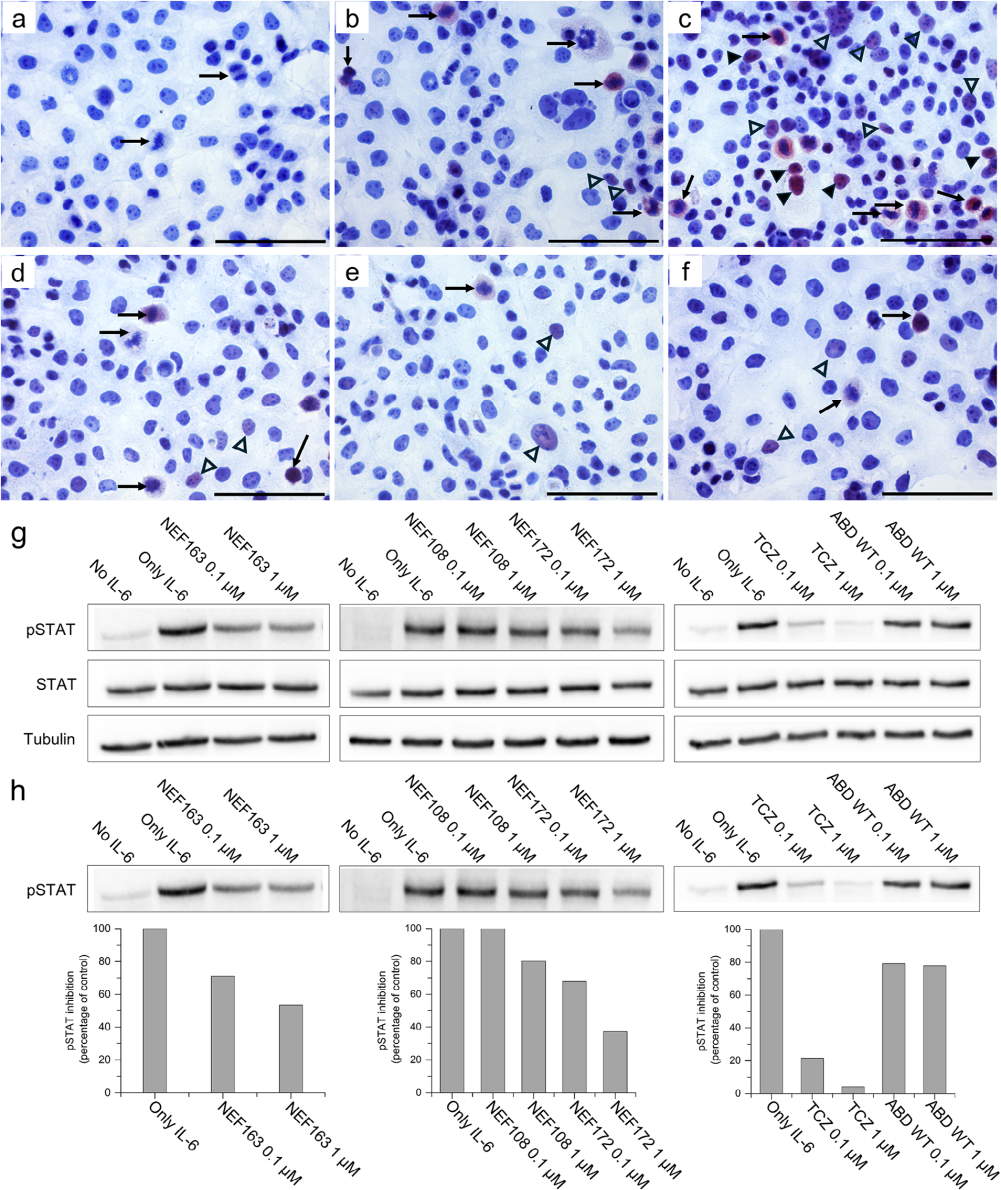
Analysis of NEF binders inhibitory activity on pSTAT3 production in carcinoma cells. (**a-f**) Immunocytochemical analysis of pSTAT3(S727) staining in pancreatic carcinoma PaTu cells; (**a**) negative control, (**b**) ABDwt pretreated cells without IL-6 stimulation, (**c**) ABDwt pretreated cells after IL-6 stimulation, (**d**) NEF108 pretreated cells after IL-6 stimulation, (**e**) NEF163 pretreated cells after IL-6 stimulation, and (**f**) NEF172 pretreated cells, after IL-6 stimulation. The magnification bar represents 100 μm. (**g, h**) Analysis of the inhibitory effect of NEF proteins on STAT3(T705) phosphorylation by Western blot on U87MG glioblastoma cells. (**g**) Cells were incubated in serum-free medium for 9 h. Afterwards, activation of pSTAT3 was carried out using hIL-6 in the presence of NEF binders for 15 min. TCZ antibody and ABDwt were used as positive and negative controls. Cells were lysed and protein concentration was calculated. The total protein amount per lane was 45 µg. Detection of bands on the membrane was performed by rabbit anti-pSTAT3(T705) mAb, anti-Stat3 mouse mAb, anti-alpha Tubulin mAb, and anti-rabbit-IgG-HRP or anti-mouse IgG-HRP conjugates, respectively. (**h**) Densitogram of the signal measured for pSTAT3(T705) in U87MG cell lysates evaluated by the ImageJ software.

To further support that NEF binders inhibit pSTAT3 production, hIL-6-stimulated pancreatic carcinoma cells (PaTu cell line) were treated in the presence or absence of NEF binders and analyzed immunocytochemically (Fig. 4). We detected an increase in pSTAT3-positive cells after hIL-6 stimulation in comparison to unstimulated cells (Fig. 4a-c). Meanwhile, PaTu cells pre-incubated with NEF proteins (NEF108, NEF163, and NEF172) and stimulated with hIL-6 comparatively showed a decrease in the number of pSTA3-positive nuclei (Fig. 4d-f). Also, we observed only a small number of cells with weak nuclear positivity for pSTAT3(S727) in unstimulated cells. In mitotic cells (examples highlighted by black arrows), STAT3(S727) phosphorylation is known as a mitosis-associated event [32]. Thus, we have excluded these mitotic cells from our observations. After stimulation by hIL-6 for 15 min, we observed an increased number of pSTAT3(S727) positive nuclei in PaTu cells, including highly positive cells (highlighted by black arrowheads), and in control cells (ABDwt-treated) (Fig. 4c). In contrast, hIL-6 treatment in pretreated PaTu cells with NEF108, NEF163, and NEF172 did not show an increase in the number of positive nuclei or intensity by comparison to the control cells (Fig. 4d-f).

To further verify the role of NEF proteins on signal transduction, we detected pSTAT3(T705) in cell lysates of hIL-6-activated U87MG cells, which are reported to be hIL-6R positive [33, 34]. In Fig. 4 g, h, Western blot data confirm that NEF variants reduce pSTAT3(T705) production in U87MG cells. We also used the TCZ antibody as a positive control and ABDwt parental non-mutated scaffold protein as a negative control (Fig. 4 g, h). The NEF172 variant was found to be the strongest inhibitor, while NEF163 and NEF108 exhibited only a moderate or weak inhibitory effect on STAT3(T705) phosphorylation (Fig. 4h). Overall, this observation demonstrates the effect of NEF proteins on the inhibition of IL-6R-mediated signal transduction.

### Effect of NEF binders on human primary dermal fibroblasts and malignant melanoma cells

**Fig. 5:**
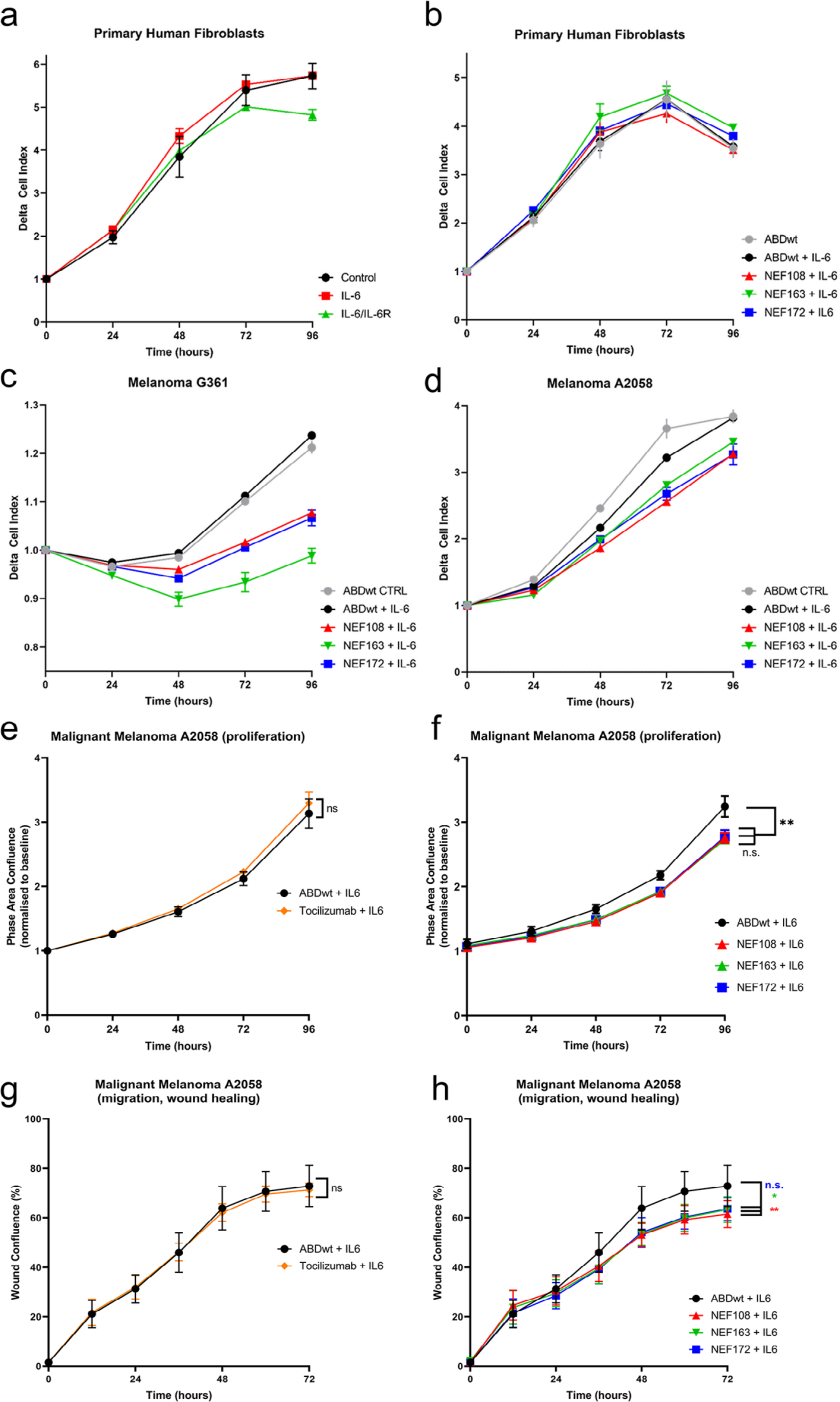
Effect of NEF binders on cell proliferation and migration. (**a-d**) Cell proliferation assay (iCELLigence). (**a**) The effect of IL-6 and chimeric proteins composed of IL-6 and IL-6R (IL-6/IL-6Rα) was compared with the growth of non-influenced control fibroblasts. (**b, c, d**) Effects of the proteins ABDwt (control), (**b, c, d**) ABDwt + IL-6, (**b, c, d**) NEF108 + IL-6, **(b, c, d**) NEF163 + IL-6, and (**b, c, d**) NEF172 + IL-6, are shown on (**a-b**) human primary fibroblasts, (**c**) melanoma cells G361, and (**d**) A2058. (**e, f**) NEF inhibitors also reduce proliferation of A2058 melanoma cells measured by automatic optical instrumentation (Incucyte) in comparison to ABDwt and monoclonal humanized TCZ antibody. (**g, h**) Scratch test measurements using Incucyte instrumentation and software. Among the three tested NEF binders, NEF108 significantly reduces the migration of A2058 melanoma cells in comparison to the clinically employed TCZ antibody. Herein, * = *p* < 0.05; ** = *p* < 0.01, and n.s. = not significant provides the statistical significance of the data

The chimeric protein composed of hIL-6 and its receptor β-mimicking the transactivation pathways of soluble hIL-6 receptors occupied with hIL-6 influenced the normal human primary fibroblast DFO35 (Fig. 5a). The proliferation of DFO35 is not affected by a high dose of LPS, as demonstrated in Figures S2 and S3. Also, the application of hIL-6 and NEF binders to DFO35 cell culture has practically no effect on their growth characteristics (Fig. 5b). On the other hand, the application of hIL-6 to the culture of both cutaneous melanoma G361 and A2058 cells exhibited a small effect on their growth characteristics (Fig. 5c, d). The NEF binders to hIL-6Rα were not toxic for both studied cell lines. Melanoma cells were sensitive to the NEF binders, but the results were cell line specific, with a higher effect on G361 cells than on A2058 cells (Fig. 5c, d). The highest efficiency was observed for binder NEF163 in G361 cells (Fig. 5c). The inhibitory effect of NEF proteins on the proliferation of A2058 cells was also confirmed by an independent method using Incucyte (Fig. 5e, f). In addition, substantial suppression of A2058 melanoma cell migration was observed using the Scratch wound healing assay by Incucyte (Fig. 5g, h).

### NEF binders suppress proliferation and migration of pancreatic cancer cells

**Fig. 6:**
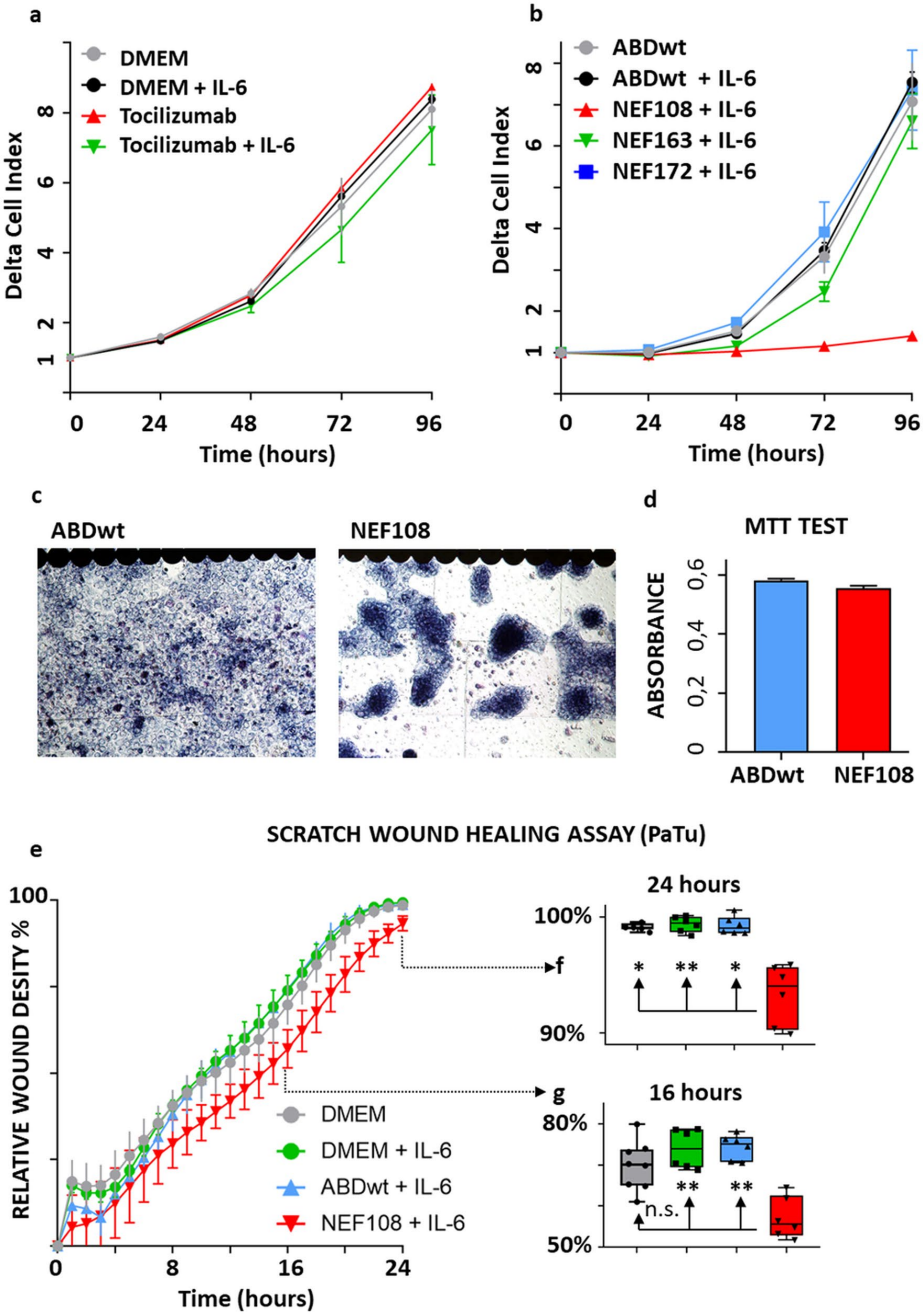
Cell proliferation assay (iCELLigence real-time cell analyzer) and Scratch test (IncuCyte). Effect of clinically approved hIL-6Rα inhibitor (**a**) Tocilizumab (TCZ) on the growth of cells of the PaTu cell line from the ductal adenocarcinoma of the pancreas in comparison to (**b**) NEF binders. (**c, d**) The high effect of NEF108 was conditioned by the inhibition of cell migration as detected by microscopy (ABDwt versus NEF108) and the MTT test. (**e-g**) The Scratch test (IncuCyte) demonstrated the inhibitory effect of NEF108 on the migration of PaTu cells. Herein, * = *p* < 0.05; ** = *p* < 0.01, and n.s. = not significant provides the statistical significance of the data

The inhibitory effect of NEF proteins on the proliferation of MiaPaCa pancreatic cancer cells was also observed (Fig. S4). The gold standard for hIL-6Rα inhibition is the humanized monoclonal TCZ antibody, which was clinically approved. The effect of supplementation of this antibody to the culture medium on the growth characteristics of the PaTu cell line (ductal adenocarcinoma of the pancreas) was observed. However, it was negligible even after stimulation by hIL-6 (Fig. 6a). The application of NEF binders to these cells was more efficient; namely, the application of the NEF108 binder induced the highest effect (Fig. 6b). The result of the MTT test and microscopic observation demonstrated that this binder is not toxic because the MTT test showed a good metabolic condition of cells based on the NADH-dependent oxidoreductase activity of mitochondrial enzymes (Fig. 6c, d). The difference in the measurements (iCELLigence) between the application of ABDwt with hIL-6 and NEF108 with hIL-6 is, therefore, conditioned by the reduced migration activity of PaTu cells after NEF108 treatment (Fig. 6c, d). The automated scratch assay (IncuCyte) supported the observation and clearly demonstrated the statistically significant anti-migratory effect of NEF108 application to PaTu cells (Fig. 6e-g).

**Fig. 7:**
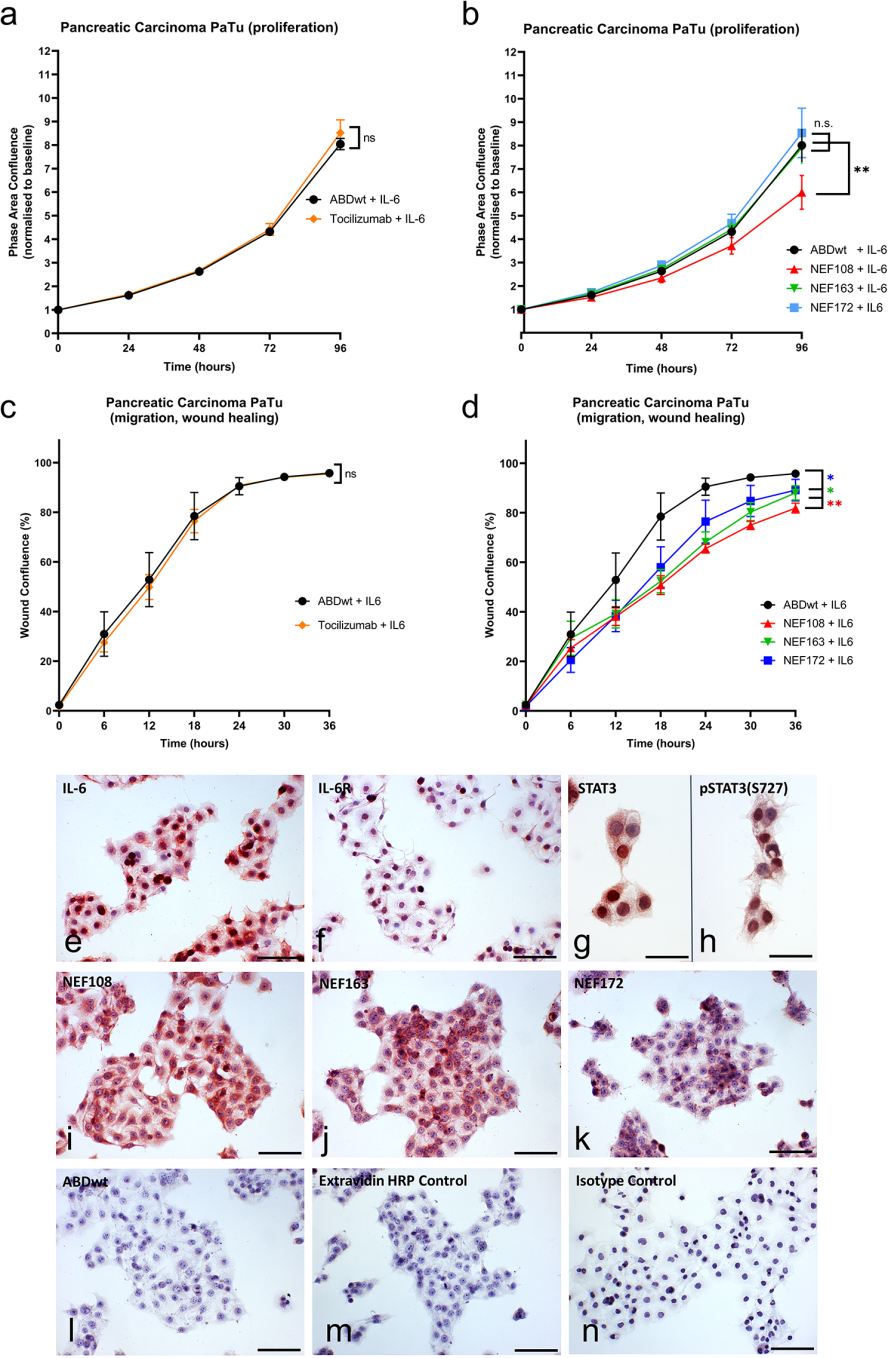
Effect of NEF blockers on PaTu cell proliferation, migration, IL-6, and IL-6R expression. (**a-b**) For the cell proliferation assay, PaTu cells per well were seeded on 96-well plates overnight. The next day, the medium was replenished for continuous screening in the (**a**) presence of ABDwt and tocilizumab (TCZ) or (**b**) NEF proteins using the Incucyte. All experiments were performed in six technical replicates (wells) using four defined points for confluence measurement every 2 h for four consecutive days. Resulting confluence was determined by Proliferation software and obtained data (in%) were analysed using GraphPad Prism (* = *p* < 0.05; ** = *p* < 0.01; n.s. = not significant, ANOVA). (**c-d**) For the migration (wound healing) assay, PaTu cells per well were seeded on 96-well plates. The next day, the medium replaced, and cells were preincubated with (**c**) ABDwt and tocilizumab (TCZ) or (**d**) NEF binders overnight. Afterthat, standardised wounds were created using Incucyte® WoundMaker and then continuously monitored using the Incucyte. All experiments were performed in six technical replicates (wells) using two defined points for wound size measurement every 2 h up to maximum three consecutive days. Resulting wound healing data was acquired using Incucyte® Scratch Wound analysis software and analysed using GraphPad (* = *p* < 0.05; ** = *p* < 0.01; n.s. = not significant, ANOVA). (**e**) In immunocytochemical staining, cells exhibited a high signal of IL-6 expression and (**f**) a lower but specific signal for IL-6 receptor expression. (**g, h**) The cells expressed STAT3 and were able to translocate pSTAT3 to the nucleus. (**i, j, k**) NEF binders, especially NEF108, strongly bind to PaTu cells, while (**l**) the missing ABDwt affinity to PaTu cells resulted in a negative staining. (**m**) Control reaction with isotype antibody and (**n**) Extravidin HRP conjugate confirm the specificity of immunohistochemical reactions

To verify the effect of NEF proteins on the proliferation of PaTu cells, we performed an independent cell proliferation assay using Incucyte (Fig. 7a-d). While we did not observe any substantial effect of TCZ on PaTu cell proliferation (Fig. 7a), we confirmed a prominent inhibition of the NEF108 variant (Fig. 7b). Similarly, TCZ had no effect on the migration of PaTu cells tested by Incucyte in the wound healing assay (Fig. 7c). In correlation to PaTu proliferation data, NEF108 exhibited the most prominent inhibitory effect on cell migration (Fig. 7d). Thus, the three selected NEF binders exhibit a considerable anti-proliferation and anti-migration effect on pancreatic cancer cells.

### Expression of IL-6 and IL-6Rα on PaTu cells and staining with NEF variants

PaTu cells strongly expressed hIL-6 (Fig. 7e), and the receptor for this cytokine (hIL-6Rα) was also detected in vitro (Fig. 7f). STAT3 (Fig. 7g), the principal downstream effector of the hIL-6 signaling pathway, was detected in cells; it was phosphorylated (on serine 727, pSTAT3) upon hIL-6 stimulation and present in nuclei (Fig. 7h). PaTu cells bind NEF variants with high affinity (NEF108 - Fig. 7i, NEF163 - Fig. 7j, and NEF172 - Fig. 7k). This contrasted with the ABDwt control, which did not bind to the cells (Fig. 7l). Negative controls for in vivo biotinylated proteins were performed using HRP-labeled Extravidin (Fig. 7m), and negative controls for antibody-based staining used isotype immunoglobulins (Fig. 7n).

### Effect of NEF binders on migration and proliferation of GAMG glioblastoma cells and binding pose prediction for NEF proteins

**Fig. 8:**
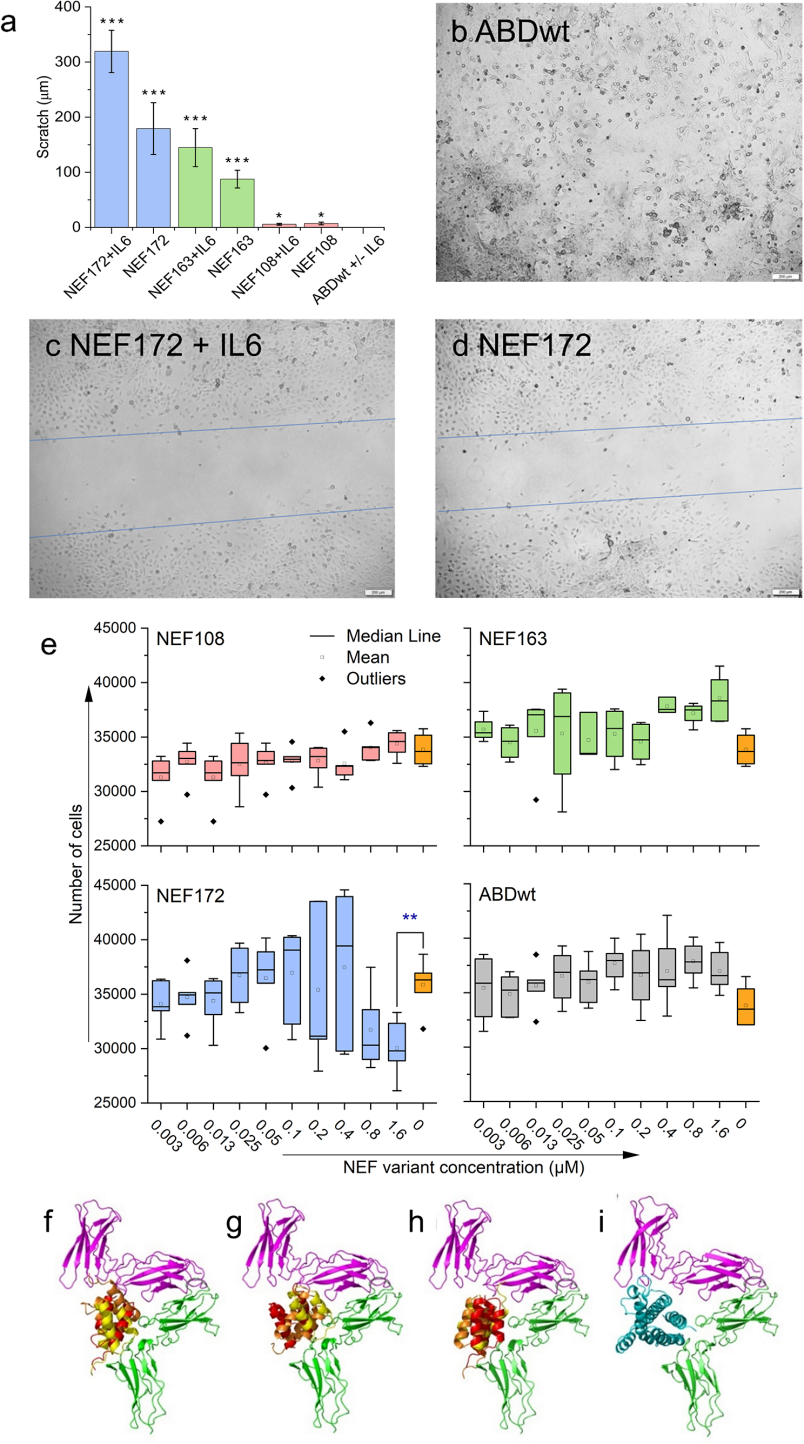
Migration and proliferation of glioblastoma GAMG cells in the presence of NEF binders and prediction of binding modes of NEF proteins by docking. (**a**) Cell migration assay evaluated using the scratch gap after 48 h of incubation with 200 nM of NEF172, NEF163, and NEF108 +/- 50 ng/ml of hIL6. (**b**) Representative image for gap evaluation of ABDwt. (**c**) Width of gap evaluation for NEF172 + IL-6. (**d**) Width of gap evaluation for NEF172 without IL-6. (**e**) Cell proliferation assay for GAMG cells evaluated with the CCK-8 kit after 24 h of incubation with different concentrations of NEF proteins and ABDwt control. The results are presented as two independent experiments performed in triplicate. (**a, e**) For statistical evaluation, the one-way ANOVA was used (* = *p* < 0.05; ** = *p* < 0.01; *** = *p* < 0.001). (**f-i**) Summary of NEF variants docking to the structure of the hIL-6Rα/hIL-6Rβ complex (pdb id 1p9m). The hIL-6Rα is shown in magenta, the hIL-6Rβ in green, the hIL-6 ligand in cyan, and the NEF variants are shown in decreasing predicted order of binding as red, orange, and yellow cartoon. (**f**) Binding poses for NEF108, (**g**) for NEF163, (**h**) for NEF172, and (**i**) shows the ternary hIL-6Rα/hIL-6Rβ complex (doi 10.5281/zenodo.10213658)

The migration assay using GAMG cells shows the highest anti-migration potential of NEF172 (in average 200 μm), followed by NEF163 (in average 100 μm). The NEF108 did not show any substantial anti-migration effect (Fig. 8a); similarly, the ABDwt control showed no scratch gap (Fig. 8b). The incubation with hIL-6 increases the anti-migration effect of both NEF172 and NEF163 proteins (Fig. 8a). In the case of NEF172, the scratch gap was increased to 300 μm, and in the case of NEF163, to 150 μm (Fig. 8a, c, d). The incubation with hIL-6 did not change the effect of NEF108 (Fig. 8a).

Additionally, the effect of NEF proteins on the proliferation of GAMG glioblastoma cells was tested. Cells were incubated with different concentrations of NEF ligands for 24 h, and cell numbers were estimated by the CCK-8 kit. Results are presented as a floating bar chart in Fig. 8e; only the variant NEF172 demonstrated an inhibitory effect on proliferation of GAMG cells at the highest concentration.

To explain the observed inhibitory function of the NEF variants, we performed binding mode prediction on IL-6Rα (Fig. 8k-m). The top three predicted binding modes for all modeled NEF variants share a common site on hIL-6Rα. A comparison of the NEF binding prediction to the existing crystal structure of the hIL-6/hIL-6Rα complex (Fig. 8n) reveals that the natural hIL-6/hIL-6Rα binding site overlaps with the predicted NEF binding modes, supporting the experimentally observed inhibitory action of NEF variants.

### Biophysical characterization of NEF binders

To estimate the thermal stability of the NEF variants (NEF108, NEF163, and NEF172), melting temperature was measured using the NanoDSF method (Fig. S5). The melting temperature of NEF variants (NEF-TolA) was compared to that of ABDwt-TolA. The melting temperature for ABDwt-TolA was 66.5 °C, which is similar to the previously reported value [35]. The melting temperatures for NEF108, NEF163, and NEF172 were 60.5 °C, 54.0 °C, and 59.3 °C, respectively (Table S3). Therefore, randomization of ABD scaffold wild-type residues caused different degrees of destabilization among NEF variants, which is expected considering the number of introduced mutations. However, the stability of the selected NEF variants remains high and meets the declared application requirement. In this study, thermal stability was tested in PBS but it can vary with buffer composition.

The far UV CD spectrum was measured to determine the secondary structure composition of NEF variants. CD spectra of NEF variants were compared with ABDwt-TolA CD spectra (Fig. S6). ABDwt-TolA is a fusion protein of three α-helical ABD domains fused with the TolA domain, which is composed of a long α-helix and α + β globule (pdb: P19934). Analysis of CD spectra for ABDwt-TolA revealed that proteins contain predominantly α-helical structures, while other secondary structure types are present in low content. In the case of the NEF variants, α-helix is still the predominant structural type in all NEF variants, but to a lesser degree compared to ABDwt, while the presence of other structural types increased. The result corresponds to the Tm values obtained. NEF108 has only slightly higher Tm according to NanoDSF and α-helical content according to CD spectra than NEF172. Similarly, NEF163 has significantly lower Tm and α-helical content compared to NEF108 and NEF172.

### Binding of NEF proteins to human peripheral blood mononuclear cells

**Fig. 9:**
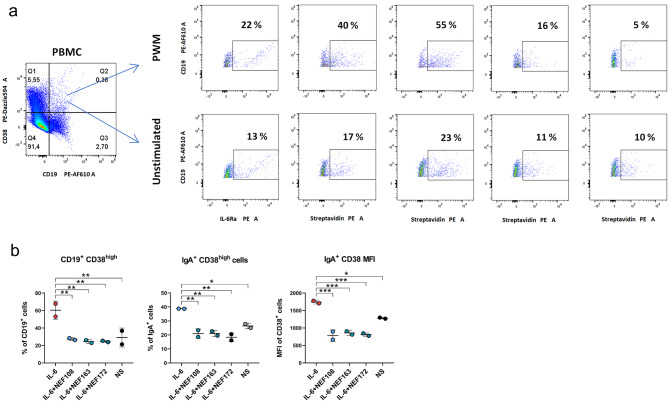
Binding of NEF proteins to primary human cells. (**a**) NEF binding to IL-6Rα expressed on stimulated primary B cells. PBMCs were stimulated for 96 h with 10 µg/ml PWM. The B cell subpopulation was stained with antibodies specific to CD19 (PE-AF610), CD38 (PE-DZL594), and with either anti-IL6Rα antibody or one of the NEF binders (NEF108, NEF163, and NEF172) and detected by flow cytometry. The gating strategy for CD19^+^ and CD38^+^ cells is shown on the left subpanel. IL-6Rα expression was determined in the gate Q2 (CD19^+^CD38^+^), corresponding to plasmablasts/plasma cells. Plots represent populations positive for individual NEF binders or anti-IL-6Rα before and after PWM stimulation. The percentage of cells positive for IL-6Rα is shown on the plots. All tested binders show a similar (NEF172) or higher (NEF108 and NEF163) percentage of IL-6Rα-positive cells in comparison with anti-IL6Rα antibody detection. ABDwt was used as an unspecific control. (**b**) NEF inhibition of IL-6-driven stimulation of B cell differentiation toward plasmablasts/plasma cells. PBMCs were stimulated by hIL-6 for 7 days with or without NEF binders. Differentiation toward plasmablasts/plasma cells was detected as CD19^+^CD38^+^ cells and their IgA^+^ subpopulation by specific fluorophore labeled mAbs by flow cytometry. *P* values were calculated using one-way ANOVA with Tukey’s post-hoc test. * = *P* < 0.05, ** = *P* < 0.01, *** = *P* < 0.001. Graphs show means and SD. NS means unstimulated control in the absence of NEF binders

To confirm the specificity of NEF binding to hIL-6Rα present on primary blood cells, PBMCs were purified by gradient centrifugation, and differentiation of B cells was induced by pokeweed mitogen (PWM) lasting for 4 days to obtain activated plasmablasts/plasma cells (CD19^+^CD38^+^) positive for hIL-6Rα [36, 37]. The results are summarized in Fig. 9. Unstimulated and PWM-stimulated PBMCs were stained with anti-CD19 and anti-CD38 antibodies and, at the same time, with either anti-IL-6Rα antibody or with one of the NEF binders, NEF108, NEF163, and NEF172, or ABDwt protein to detect the presence of the hIL-6Rα receptor. Flow cytometry data show that all three NEF binders, NEF163, NEF172, and NEF108, are able to recognize hIL-6Rα with the same (NEF172) or better (NEF163 and NEF108) ability than anti-IL6Rα antibody (Fig. 9a).

Further, we tested the ability of NEF binders to inhibit IL-6-mediated in vitro activation and differentiation of B cells within the PBMC population toward plasmablasts/plasma cells. As shown in Fig. 9b, left panel, B cells differentiate toward a population of plasmablasts/plasma cells, reaching 60% of total CD19^+^ cells. In contrast, hIL-6 induced PBMCs simulation in the presence of NEF binders (NEF108, NEF163, and NEF172) significantly reduced the activation toward plasmablasts/plasma cells comparable to the levels in nonstimulated controls. In addition, we tested the ability of NEF binders to inhibit IL-6-induced PBMCs differentiation toward IgA1^+^ plasmablasts/plasma cells, as we reported earlier [38, 39]. Similarly, for the total population of plasmablasts/plasma cells, NEF binders inhibited IgA^+^ subset activation toward plasmablasts/plasma cells (Fig. 9b, middle panel). We also measured the mean fluorescence intensity (MFI) of the CD38 marker, which is substantially enhanced upon activation toward plasmablasts/plasma cells. Even here, NEF binders inhibited hIL-6-mediated activation (Fig. 9b, right subpanel) of the IgA^+^ subset.

### NEF108 protein significantly alleviates histomorphological markers of large intestine alterations in DSS colitis model

**Fig. 10:**
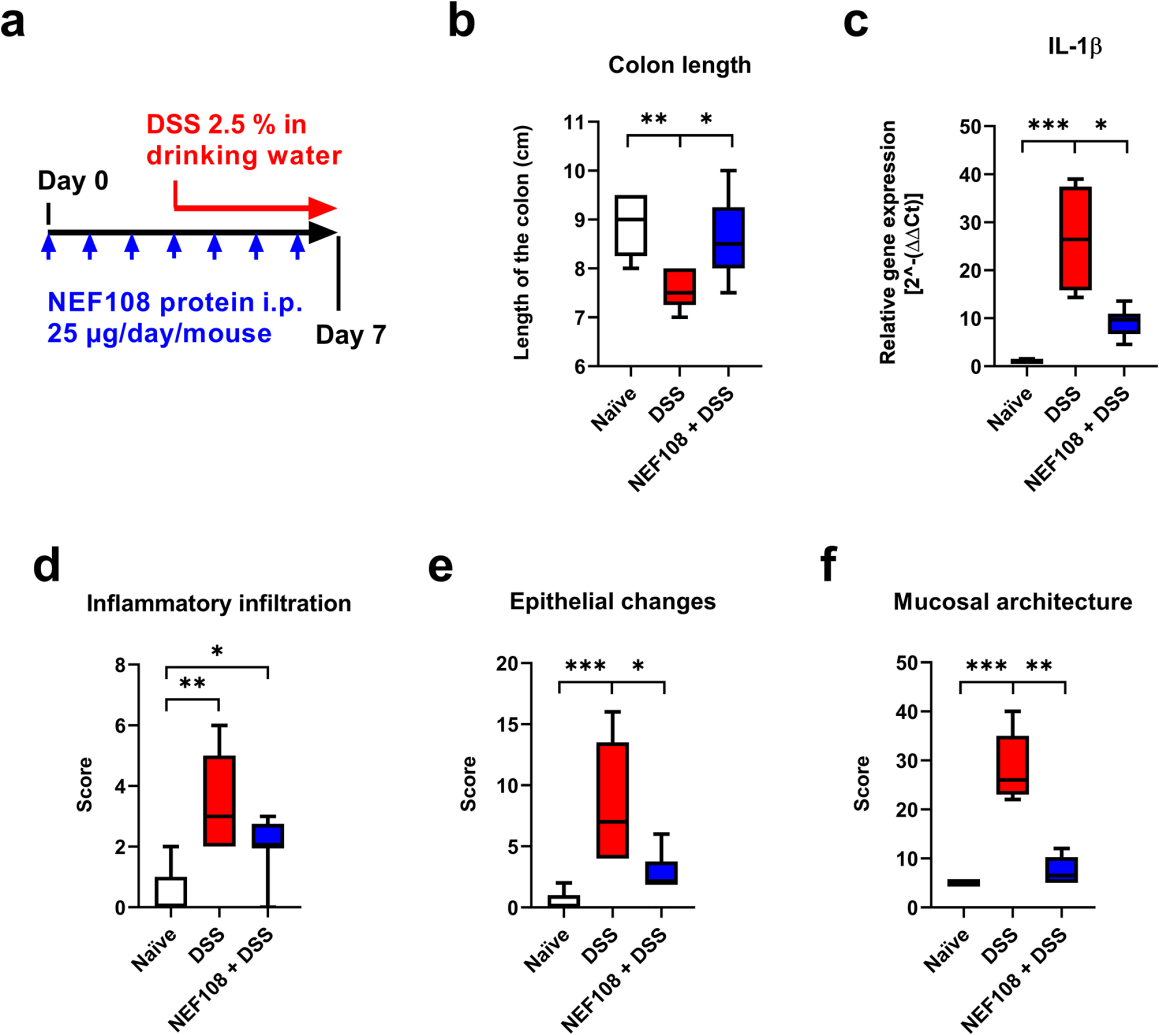
Protective effect of NEF108 ligand targeting IL-6Rα in murine model of DSS-induced colitis. NEF108 ligand in preventative-therapeutic regime was tested in the model of DSS-induced colitis. (**a**) NEF108 protein was administered daily by i.p. route (blue arrow) starting three days before the administration of DSS in drinking water (red arrow). The NEF108 application continued in parallel to DSS for subsequent 4 days. After experiment termination, (**b**) the length of the colon was determined (length between caecum and rectum) – naïve group (8.9 ± 0.4 cm) and DSS group (7.4 ± 0.4 cm) in DSS mice. NEF108-treated DSS exposed mice exhibited the colon length 8.3 ± 0.7 cm. (**c**) Colon IL-1β transcript level was measured by RealTime-PCR. Histological classification was assessed for (**d**) inflammatory cell infiltrate, (**e**) epithelial changes, and (**f**) mucosal architecture. In the case of inflammatory infiltrate and epithelial changes, the effect of NEF108 was observed in approximately 50% of mice. Statistical differences were analyzed by Kruskal-Wallis one-way ANOVA followed by Dunn´s multiple comparisons test. Means with SD are shown (* = *P* < 0.05, ** = *P* < 0.01, *** = *P* < 0.001)

We tested the effect of NEF108 binder in preventative-therapeutic regime of DSS colitis by assessment the colon length and histomorphological changes, namely inflammatory cell infiltration of large intestine mucosa, epithelial changes, and mucosal architecture (Fig. 10). NEF108 significantly prevented DSS-induced colon length reduction (*p* < 0.05), significantly protected colon from the mucosal architecture alterations (ulcerations, granulation tissue, irregular crypts, crypt loss, and villous blunting) (*p* < 0.01), and significantly protected from epithelial changes (Goblet cells loss, epithelial hyperplasia, cryptitis, and crypt abscesses) (*p* < 0.01), (Fig. 10b, d, e, f). Inflammatory cell infiltrate remains without significant difference between groups drinking DSS with and without NEF108 protein administration. However, we can see less significant inflammatory cell infiltrate regarding to the naïve group of mice. Furthermore, we compared the IL-1β cytokine expression as a marker of inflammatory response to DSS. NEF108 treatment significantly reduced DSS-induced IL-1β expression (Fig. 10c).

## Discussion

Cancer cells are not alone, but they represent an integral component of a highly complex ecosystem with non-cancer cells such as cancer-associated fibroblasts and immune cells [40]. The success of cancer cells depends not only on genetic alteration but it is also influenced by intercellular coordination, where cancer cells communicate with non-cancer elements through intercellular contacts or via the production of extracellular matrix or growth factors, cytokines, and/or chemokines, as well as by extracellular vesicles [41]. Interestingly, many of them have an inflammation-supporting effect. Recently, much attention has been given to the pleiotropic cytokine IL-6 because of its ability to either promote or, more rarely, inhibit tumor growth [42]. Activation of the JAK2/STAT3 signaling pathway by IL-6 has been reported to mediate tumorigenesis via regulation of key cellular processes, including apoptosis, cycle progression, proliferation, invasion, migration, metastasis, angiogenesis, and tumor cell escape from the immune system [43], as well as involvement in cancer cachexia [44], and promoting the process of epithelial-mesenchymal transition (EMT) and stem cell-like features [45]. The regulation and inhibition of the IL-6/JAK2/STAT3 pathway is conducive to cancer prevention and treatment as well as improved prognosis and, therefore, represents an important target for designing anti-cancer drugs [46, 47].

Cancer-associated fibroblasts (CAFs) are an essential component in the microenvironment of solid tumors, such as pancreatic carcinomas, and their composition changes with cancer progression [48] and metastasis [49]. A subgroup of CAFs, so-called iCAFs, are strong producers of inflammation-supporting factors, including IL-6 [50]. Blocking of the IL-6-mediated JAK2/STAT3 pathway could substantially suppress the proliferation and promote the apoptosis of glioma cells [51]. In support, in vitro blocking of IL-6R inhibits cell proliferation, invasion, and neuroglobular formation of glioma tumors [52]. Also, IL-6 trans-signaling is constitutively active in several pancreatic cancer (PC) cell lines [53]. Thus, in vitro blocking of IL-6R signaling by TCZ showed pSTAT3 downregulation and inhibition of IL-6 expression in both pancreatic cancer cells and mesenchymal stem cells (MSCs) [54]. Also, enhanced IL-6 expression was positively correlated with lymph node metastasis, tumor differentiation, and vascular invasion in PC patients [55, 56].

Based on experimental data, therapy focused on IL-6/STAT3 signaling should be a suitable target for anti-cancer therapy because it can also influence other aspects of malignant disease, such as wasting and depression. Unfortunately, as demonstrated in the therapeutic application of TCZ and other anti-IL-6 signaling drugs, their anti-cancer effect was not prominent [57]. The more perspective should be their use in combination with other anti-cancer drugs or the development of new blockers preventing interactions of IL-6 with the receptor complex. Our data demonstrate that NEF proteins (NEF108, NEF163, and NEF172) compete with IL-6 cytokine for binding to IL-6Rα in ELISA (Fig. 1b) as well as on the cell surface of *hIL-6Rα*-transfected HEK293T cells (Fig. S7). However, none of these NEF variants compete with TCZ antibody, as tested by LigandTracer method (Fig. S8, S9). Small NEF proteins, thus, should be suitable candidates for blocking IL-6 signaling because they seem to be more efficient, at least in certain cell types, than the golden standard, such as TCZ antibody, under in vitro conditions. Also, the effect of NEF proteins on normal fibroblasts is negligible, in contrast to the effect of the fusion protein IL-6/IL-6 receptor on IL-6 transactivation activity. This observation harmonizes with the data of others [58], which can be interpreted by the low expression of IL-6 receptors in fibroblasts [59] as well as documented in The Human Protein Atlas (https://www.proteinatlas.org/ENSG00000160712-IL6R).

Interestingly, the inhibitory efficiency of NEF variants varied in different cell lines. NEF163 had the most prominent effect on the proliferation of both the melanoma cell lines G361 and A2058. In the case of pancreatic cell lines, all three NEF variants had a limited effect on proliferation. However, NEF108 considerably restricted the migration of the PaTu cell line. NEF172 had the most prominent inhibitory effect on GAMG cell migration, while NEF108 and NEF163 had a weaker or no effect, respectively. These observed differences in the binding of NEF variants to cell surface hIL-6Rα could be caused by several factors. According to the NCBI and UniProt databases, there are hundreds of single nucleotide polymorphisms (SNPs) in the human *IL-6Rα* gene that are known for amino acid substitutions. However, little is known about their biological effect on the hIL-6Rα function. We hypothesize that these SNPs are cell line-specific, thus affecting the affinity or accessibility of the cytokine binding site for particular NEF protein variants.

To characterize NEF interaction with IL-6Rα on primary cells, we used the B cell (CD19^+^) subpopulation of PBMCs. It was formerly reported that non-stimulated B cell populations do not express detectable levels of IL-6Rα (CD126), whereas stimulated B cell populations, including plasmablasts and early plasma cells, are IL-6Rα positive [60]. As expected, comparison of populations before and after PWM stimulation confirmed an increase in the number of NEF-stained cells analogously to anti-IL-6Rα mAb staining (Fig. 9a). This observation supports NEF binders’ ability to recognize IL-6Rα in primary human cells. Furthermore, we assessed the biological function of NEF binders as potential inhibitors of IL-6 signaling on primary B cells. We followed our previous reports indicating that IL-6 could substantially contribute to B cell maturation, particularly of the IgA^+^ B cell subpopulation, toward plasmablasts (CD38^+^) [39]. Here we confirmed that all three tested NEF108, NEF163, and NEF172 binders significantly reduced the population of CD38^+^ after IL-6 stimulation (Fig. 9b), indicating NEF proteins’ ability to effectively interfere with IL-6 signaling.

Additionally, we verified the inhibitory potential of NEF108 protein on the mouse version of the IL-6 receptor using murine model of DSS-induced colitis. As shown in Fig. 10, NEF108-treated mice demonstrated a significant reduction in inflammation-induced tissue damage along with the suppression of the IL-1β cytokine expression, as a marker of inflammatory response to DSS. Although this study needs to be further extended, our preliminary results strengthen the antagonistic effectiveness of NEF proteins demonstrated in vitro.

## Conclusions

Collectively, the generated NEF binders represent a promising class of new IL-6R protein antagonists that can be instrumentalized to achieve an efficient migrastatic anti-cancer treatment. In addition, NEF binders can be further characterized for their IL-6R-blocking function in autoimmune diseases such as IgA nephropathy.

### Electronic supplementary material

Below is the link to the electronic supplementary material.

Supplementary Material 1

## Data Availability

No datasets were generated or analysed during the current study.

## Abbreviations

ABD: Albumin-binding domain
Akt: protein kinase B
BSA: Bovine Serum Albumin
CAFs: cancer-associated fibroblasts
CCK-8: Cell counting kit-8
CD: Circular dichroism
DMEM: Dulbecco’s modified Eagle’s medium
DMSO: Dimethyl sulfoxide
E. coli: *Escherichia coli*
ELISA: Enzyme-linked immunosorbent assay
FBS: Fetal bovine serum
FCS: Fetal Calf Serum
FDA: Food and Drug Administration
FITC: Fluorescein isothiocyanate
gp130: glycoprotein 130
HBSS: Hank’s Balanced Salt Solution
HEPES: 4-(2-Hydroxyethyl)piperazine-1-ethanesulfonic acid
HRP: Horseradish peroxidase
IgG1: Immunoglobulin G1
IL-6: Interleukin-6
IL-6R: Interleukin-6 receptor
IL-6Rα: Interleukin-6 receptor alpha
IMAC: Immobilized metal affinity chromatography
IPTG: Isopropyl β-d-1-thiogalactopyranoside
JAK: Janus kinase
mAb: Monoclonal antibody
MAPK: Mitogen-activated protein kinase
MFI: Mean fluorescence intensities
MTT: 3-(4,5-Dimethylthiazol-2-yl)-2,5-Diphenyltetrazolium Bromide
NIR: Near-infrared
pAb: polyclonal antibody
PBMCs: Peripheral blood mononuclear cells
PC: *Pancreatic cancer*
PE: Phycoerythrin
PFA: Paraformaldehyde
PI3K: phosphoinositide 3-kinase
RBS: Ribosome binding site
SFK: Src Family Kinases
STAT: signal transducer and activator of transcription
T7p: Bacteriophage T7 RNA Polymerase Promoter
TCZ: Tocillizumab
TMB: 3,3’,5,5’-Tetramethylbenzidine
TME: Tumor microenvironment
YAP: Yes-associated protein
