# Supplementary material for: Small protein blockers of human IL-6 receptor alpha inhibit proliferation and migration of cancer cells
Source: Cell Commun Signal. 2024 May 7;22:261. doi: 10.1186/s12964-024-01630-w (PMC11075285; doi:10.1186/s12964-024-01630-w)
Supplement: Supplementary file 1 — Supplementary Material 1 [file 12964_2024_1630_MOESM1_ESM.docx]

**Supporting Information**

**Small protein blockers of human IL-6 receptor alpha inhibit proliferation and migration of cancer cells**

Yaroslava Groza^1^, Lukáš Lacina^2,3#^, Milan Kuchař^1^, Leona Rašková Kafková^4^, Kateřina Zachová^4^, Olga Janoušková^5^, Radim Osička^6^, Jiří Černý^7^, Hana Petroková^1^, Joanna Maria Mierzwicka^1^, Natalya Panova^1^, Petr Kosztyu^4^, Kristýna Sloupenská^4^, Jan Malý^5^, Jozef Škarda^8^, Milan Raška^4^, Karel Smetana, Jr.^2^, Petr Malý^1#^

^1^Laboratory of Ligand Engineering, Institute of Biotechnology of the Czech Academy of Sciences, BIOCEV Research Center, Prumyslova 595, 252 50 Vestec, Czech Republic

^2^[Institute of Anatomy,](http://anat.lf1.cuni.cz/english/index.php) 1^st^ Faculty of Medicine, Charles University, U Nemocnice 3, 12800 Prague 2, Czech Republic

^3^Department of Dermatovenerology, 1^st^ Faculty of Medicine, Charles University, U Nemocnice 2, 12000 Prague 2, Czech Republic

^4^Department of Immunology, Faculty of Medicine and Dentistry, Palacky University Olomouc and University Hospital Olomouc, Hněvotínská 3, 779 00, Olomouc, Czech Republic

^5^Centre of Nanomaterials and Biotechnologies, University of J.E. Purkyně v Ústí nad Labem, Pasteurova 3632/15, 400 96 Ústí nad Labem, Czech Republic

^6^Laboratory of Molecular Biology of Bacterial Pathogens, Institute of Microbiology of the Czech Academy of Sciences, Vídeňská 1083, 14220 Prague, Czech Republic

^7^Laboratory of Structural Bioinformatics of Proteins, Institute of Biotechnology of the Czech Academy of Sciences, BIOCEV Research Center, Prumyslova 595, 252 50 Vestec, Czech Republic

^8^Department of Clinical and Molecular Pathology, Faculty of Medicine and Dentistry, Palacky University Olomouc, Olomouc, Czech Republic, Hněvotínská 3, 779 00, Olomouc, Czech Republic

**^#^Authors for correspondence:**

Petr Malý, Institute of Biotechnology of the Czech Academy of Sciences, Prumyslova 595, 252 50 Vestec, Czech Republic; Tel. no: +420 325873763, E-mail: [petr.maly@ibt.cas.cz](mailto:petr.maly@ibt.cas.cz)

Lukáš Lacina, [Institute of Anatomy,](http://anat.lf1.cuni.cz/english/index.php) 1^st^ Faculty of Medicine, Charles University, U Nemocnice 3, 12800 Prague 2, Czech Republic. Tel. no: +420 224965873, E-mail: lukas.lacina@lf1.cuni.cz

**Results**

**Table S1. Ribosome display selection conditions**

| Cycle number | 1 | 2 | 3 |
| --- | --- | --- | --- |
| Immobilized IL-6Rα, µg/ml | 25 | 25 | 25 |
| Tween20 in Wash buffer (%) | 0.05 | 0.05 | 0.25 |
| Wash times | 5 | 10 | 10 |


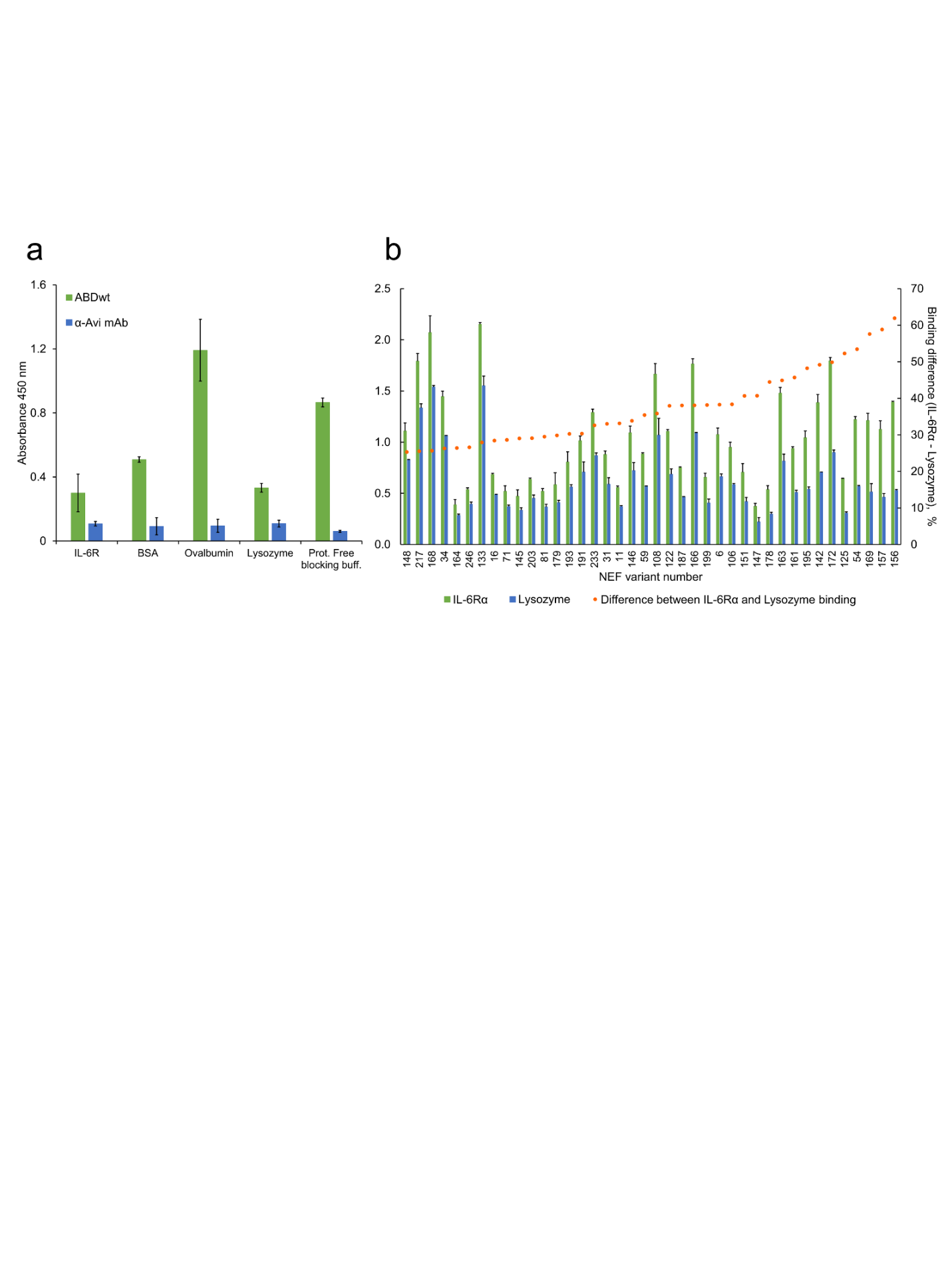


**Fig. S1. a. Comparing ABDwt binding to hIL-6Rα in comparison to different irrelevant proteins in ELISA.** hIL-6Rα, BSA, ovalbumin, and lysozyme were immobilized to MaxiSorp plate. ABDwt was produced in *E. coli* BL21 (DE3) host and purified with Ni-NTA affinity chromatography. ABDwt at a concentration of 10 nM was added to immobilized proteins and MaxiSorp surface blocked with protein free blocking buffer. ABDwt was detected with anti-Avi-Tag mouse mAb and anti-mouse mAb-HRP. After incubation of HRP with TMB substrate, the reaction was stopped with 2 M H_2_SO_4_ and absorbance at 450 nm wavelength was measured. ABDwt showed low background signal when added to hIL-6Rα immobilized to MaxiSorp. Alternatively, high background signal occurred when ABDwt was added to MaxiSorp coated with BSA and ovalbumin or blocked with buffer. ABDwt added to lysozyme demonstrated background signal comparable to hIL-6Rα. Consequently, lysozyme can be used as an irrelevant protein for NEF variants screening in ELISA as it wouldn’t introduce any bias to the assay. **b. ELISA screening of the NEF library enriched with hIL-6Rα binders as a result of ribosome display selection.** hIL-6Rα and lysozyme were immobilized to MaxiSorp plate. NEF variants were produced in *E.coli* BL21 (DE3) host and added in a form of bacterial lysates. NEF variants were detected with α-Avi-Tag mouse mAb and α-mouse mAb-HRP. After incubation of HRP with TMB substrate, the reaction was stopped with 2 M H_2_SO_4_ and absorbance at 450 nm wavelength was measured. Binding of NEF variants to hIL-6Rα is depicted as pink bars, while binding to lysozyme – as blue bars. All bars represent average of a duplicate with standard deviation. Difference between NEF variant binding to IL-6Rα and lysozyme is depicted as orange dots and is used to compare specificities of the different NEF variants. It was calculated as a difference between IL-6Rα, and lysozyme absorbance values expressed in %, when the absorbance value for lysozyme binding is calculated as a percentage of the absorbance value for IL-6R, which is considered 100%. Only 40 best NEF binders are depicted.

**Table S2. Amino acid sequences of NEF variants with inhibitory potential.** Fragment of the ABD scaffold that contains randomized residues is depicted. Amino acids highlighted in blue were randomized. Amino acids that are marked red are mutations in non-randomized parts of the scaffold.

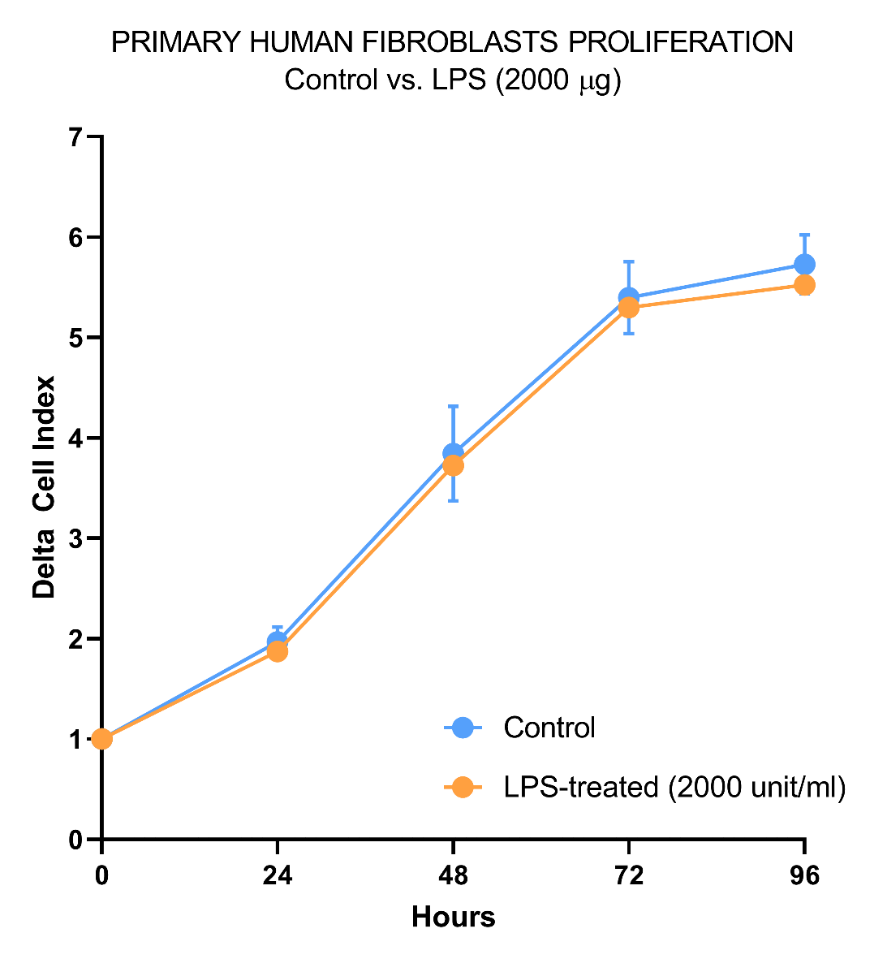


**Figure S2. Comparison of the effect of lipopolysaccharide on the proliferation of normal primary human dermal fibroblasts.** Cell proliferation was observed over 96 hours in the control medium and with LPS. No differences due to lipopolysaccharide treatment were observed in tested fibroblasts. Error bars represent standard deviation (n=2 technical replicates).


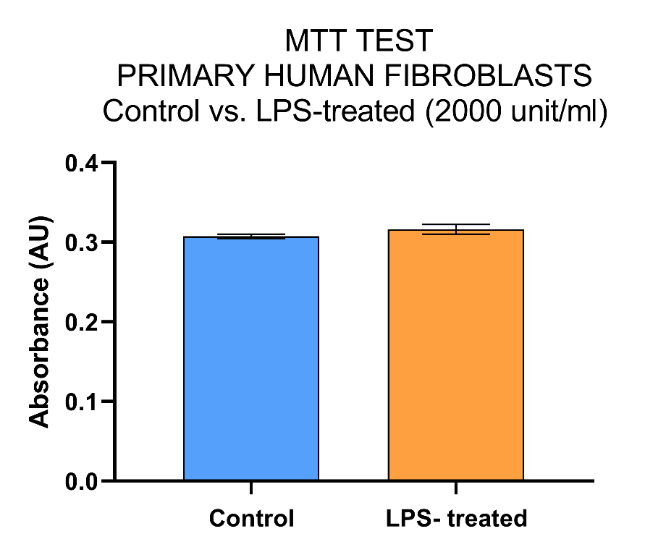


**Figure S3. Comparison of control and lipopolysaccharide treated human primary dermal fibroblasts measured by MTT test.** No differences between control and lipopolysaccharides-treated fibroblasts were observed. Error bars represent standard deviation (n=6 technical replicates).

**
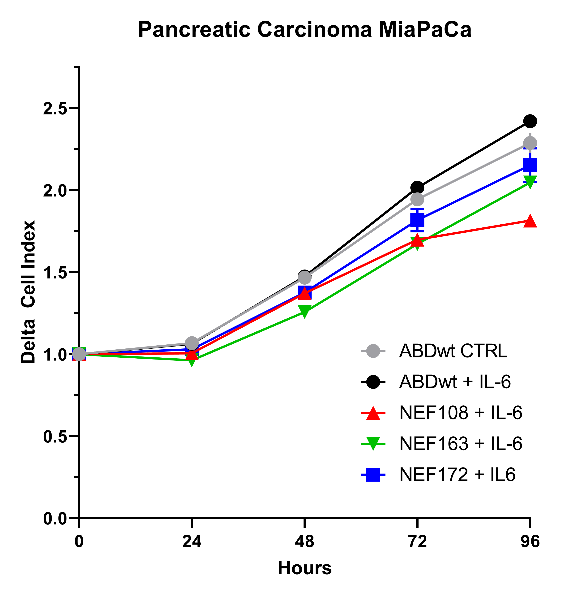
**

**Figure S4. Effect of NEF proteins on proliferation of pancreatic cells of MiaPaCa line**. NEF proteins, especially NEF108 have an inhibitory effect on cells. Error bars represent standard deviation (n=2 technical replicates).

**Table S3. Measuring of thermal stability of NEF variants using differential scanning fluorometry.** Temperature melting points of NEF proteins were determined using NanoDSF.

| **NEF protein** | | **Tm** |
| --- | --- | --- |
| **NEF108** | 60.5°C | |
| **NEF163** | 54.0°C | |
| **NEF172** | 59.3°C | |
| **ABDwt** | 66.5°C | |

**
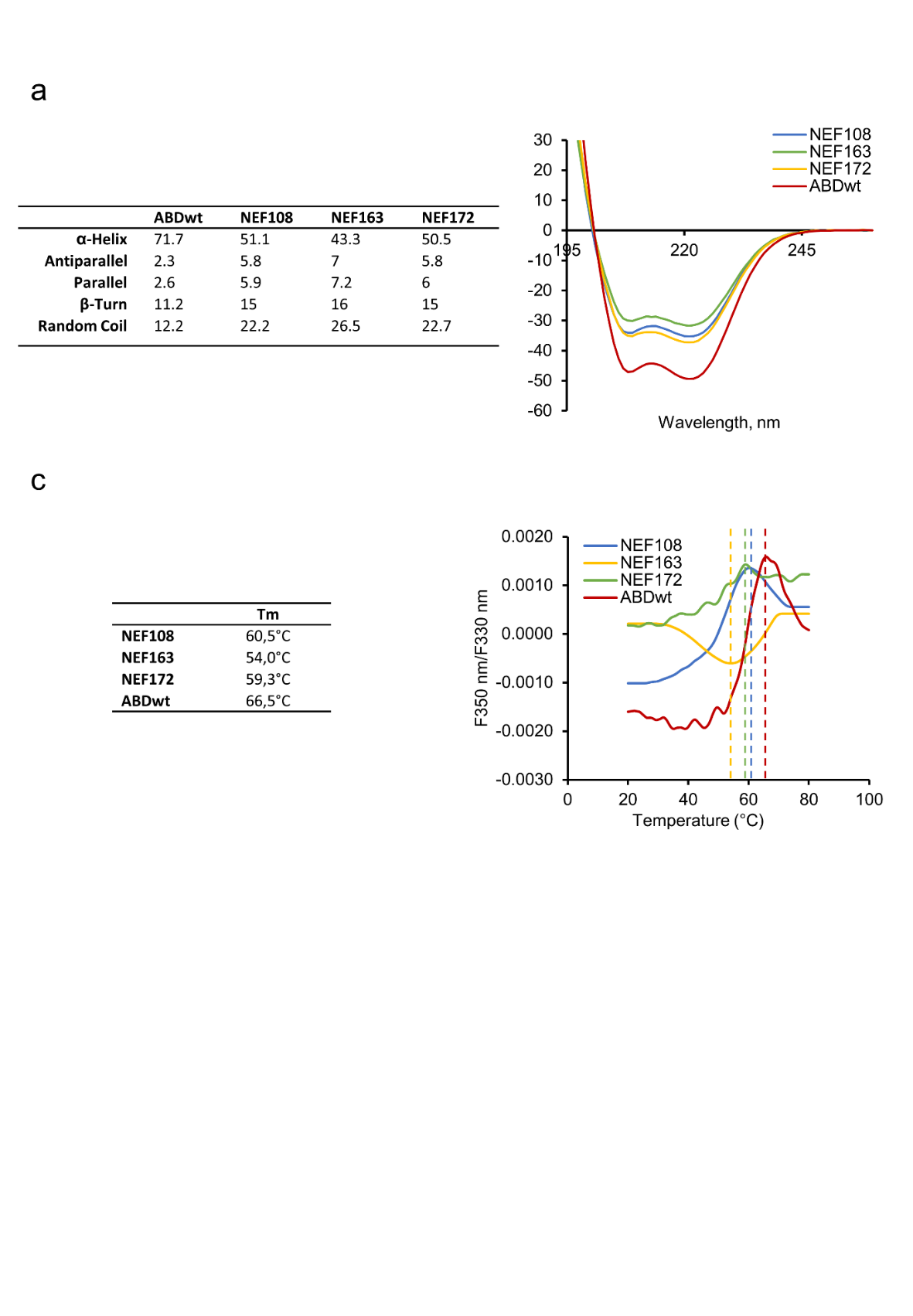
**

**Figure S5. Tryptophan and tyrosine residues of NEF variants undergo fluorescence shift upon protein melting with gradual temperature increase.** Three NEF variants (NEF108, NEF163, NEF172), as well as ABDwt, were diluted in PBS to a concentration 500 µg/ml. Protein samples were loaded into Prometheus Standard Capillaries. Samples were heated from 20 to 80 °C at a rate of 1 °C/min. Tryptophan and tyrosine fluorescence intensity was measured at 330 nm and 350 nm. Graph represents first derivative of fluorescence intensity at 350 nm and 330 nm ratio as a function of temperature. Temperature at which fluorescence shift occurs is represented as a dashed line and corresponds to melting temperature of a protein. Melting temperature values of NEF variants are shown in Table S3.

**
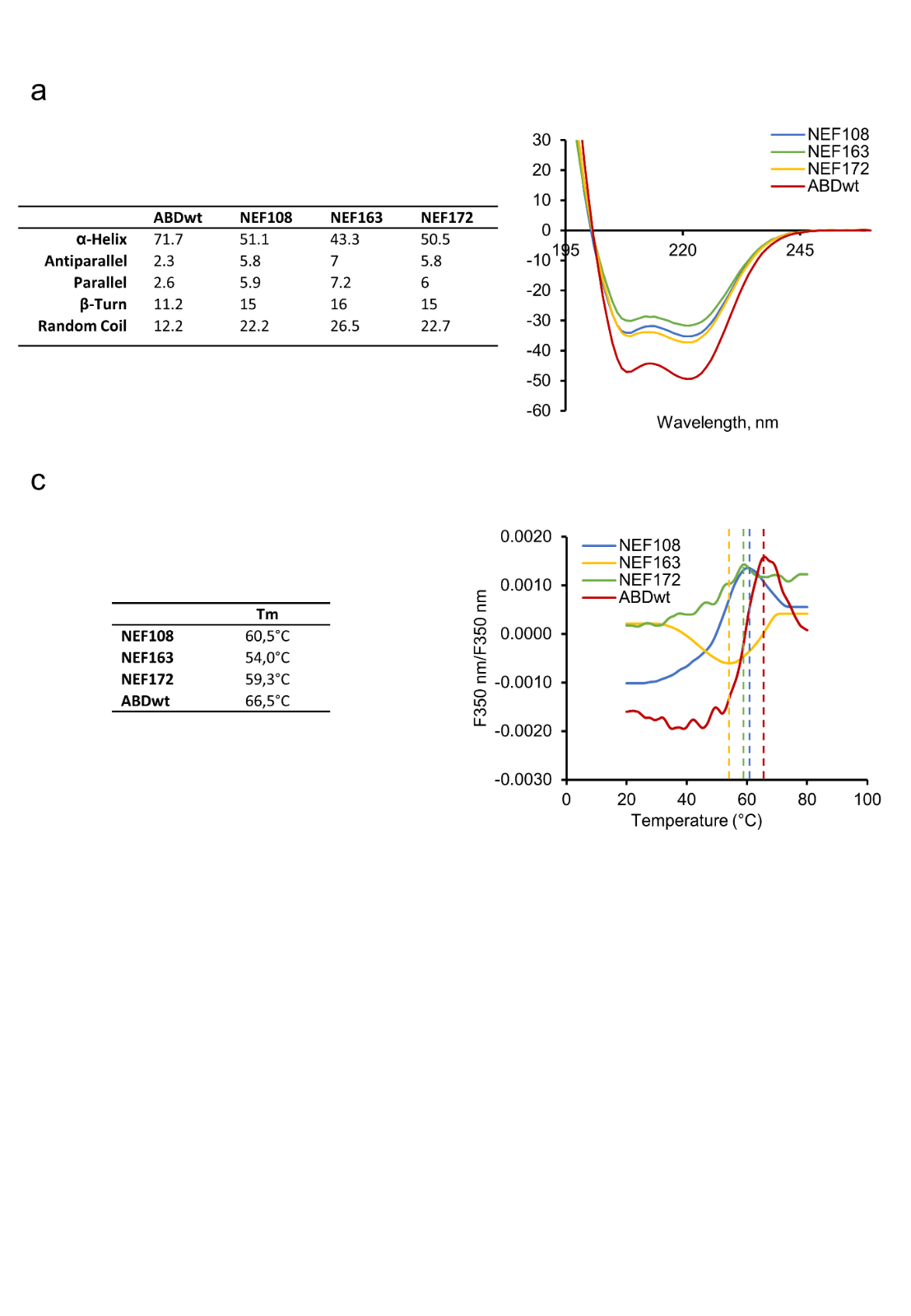
**

**Figure S6. Verification of secondary structure of NEF variants using CD spectrophotometry.** NEF proteins and ABDwt control were diluted in PBS to a concentration 200 µg/ml. Far UV CD spectra were measured in quartz cuvette at RT using Chirascan Plus spectrometer. Resulting curves are buffer-subtracted. ABDwt and NEF variants contain mostly α-helical secondary structures. Amino acid substitution in NEF variants resulted in a slight change of the protein structure comparing to ABDwt. However, it is probable that NEF variants preserve three-helical fold of the ABD scaffold.


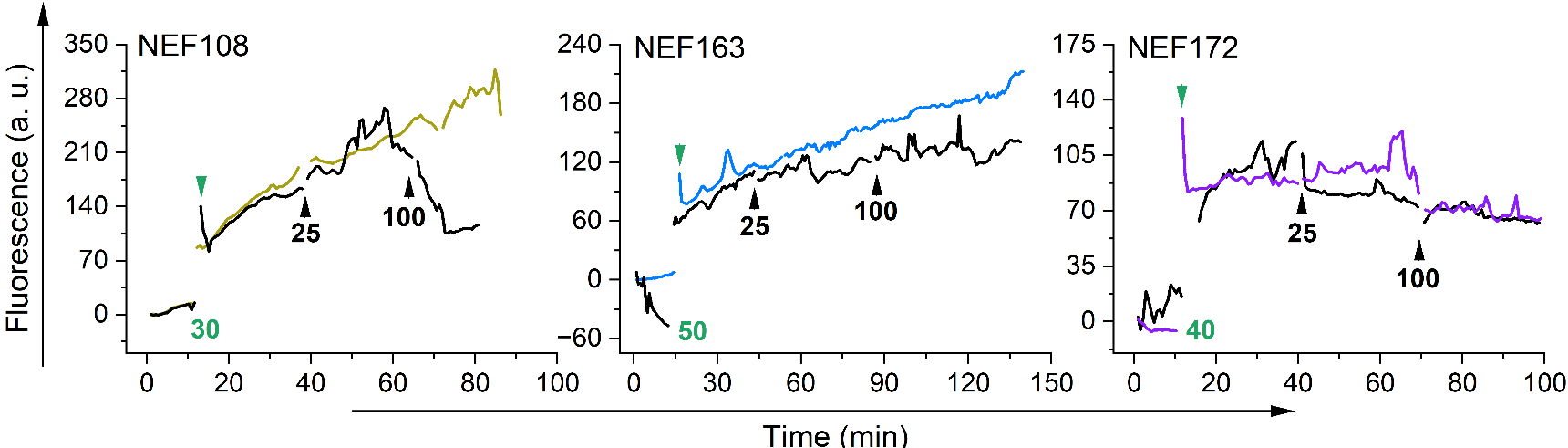


**Figure S7. Competition Ligand Tracer on hIL-6R-transfected HEK293T cells for NEF proteins with IL-6 cytokine.** Binding of NEF108, NEF163, and NEF172 to human IL-6R expressed on HEK293T transfected cells was measured in the presence or absence of increasing concentrations of IL-6. The association phase was achieved by addition of NEF variants: 30 nM for NEF108, 50 nM for NEF163 and 40 nM for NEF172 to the cell medium (shown as green arrows). Two increasing concentrations of IL-6 (25 and 100 nM) were added during the association (shown as black arrows). Black lines present the signal from cells treated with NEF variants with addition of rising concentrations of IL-6 and signal from the cells treated with NEF variants in the absence of IL-6 is depicted in different colors.


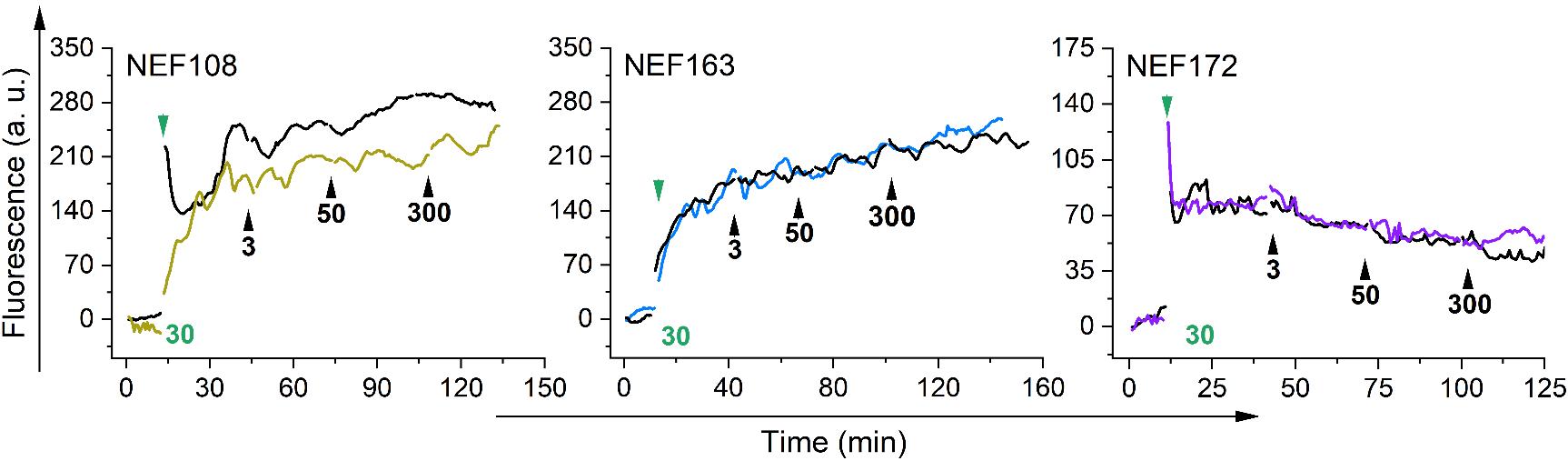


**Figure S8. Competition Ligand Tracer on hIL-6R-transfected HEK293T cells for NEF proteins with tocilizumab.** Binding of NEF108, NEF163, and NEF172 to human IL-6R expressed on HEK293T transfected cells was measured in the presence or absence of increasing concentrations of Tocilizumab. Start of the association phase was achieved by addition of 30 nM of NEF variants to the cell medium (shown as green arrows). Three increasing concentrations of Tocilizumab (3, 50 and 300 nM) were added during the association phase (shown in black arrows). Black lines present the signal from cells treated with NEF variants with addition of rising concentrations of Tocilizumab, and signal from the cells treated with NEF variants in the absence of Tocilizumab is depicted in different colors.


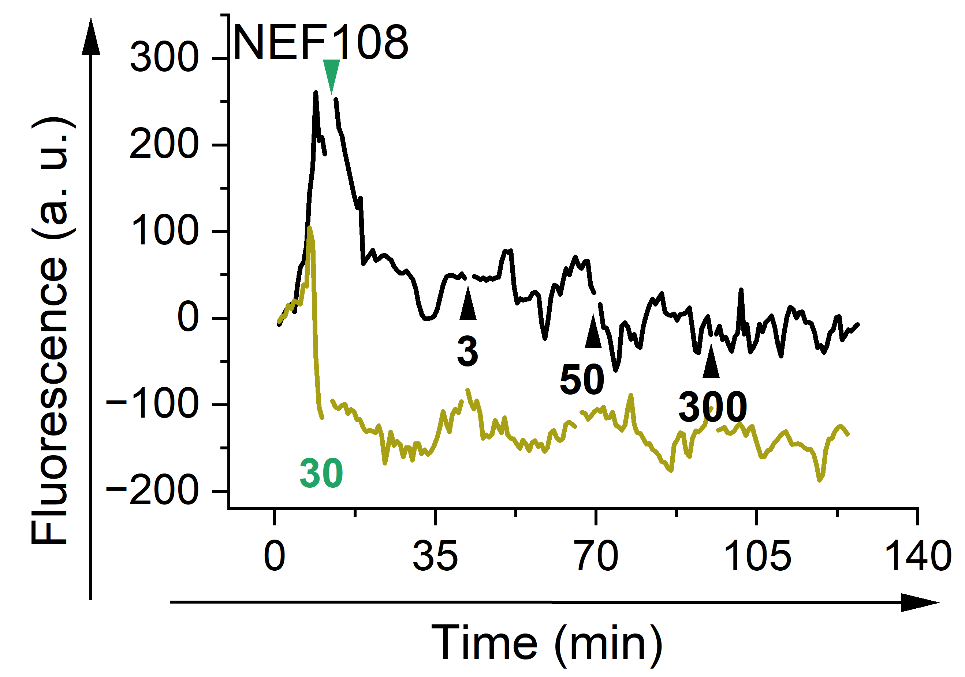


**Figure S9. Competition Ligand Tracer on non-transfected HEK293T cells.** The assay was performed on non-transfected HEK293T cells (not expressing IL-6R, serving as a negative cell line) by treatment with 30 nM of NEF108 (marked as green arrow) and measurements were performed in the presence or absence of Tocilizumab (presented as black and green lines, respectively). Black arrows show when increasing concentrations of Tocilizumab (3, 50 and 300 nM) were added.

**Table S4.** Scoring parameters and assessed values^1^

| **Inflammatory infiltrate** | Severity | Leukocyte density of lamina propria area infiltrated in evaluated hpf: | 1-4 |
| --- | --- | --- | --- |
|  | Extent | Expansion of leukocyte infiltration: | 1-3 |
| **Epithelial changes** | Hyperplasia | Increase in epithelial cell numbers per crypt; visible as crypt elongation: | 1-5 |
|  | Goblet cell loss | Reduction of goblet cell numbers relative to baseline | 1-4 |
|  | Cryptitis | Neutrophils between crypt epithelial cells | 2-3 |
|  | Crypt abscesses | Neutrophils in crypt lumen | 3-5 |
|  | Erosion | Loss of surface epithelium | 1-4 |
| **Mucosal architecture** | Ulceration | Epithelial defect reaching beyond muscularis mucosae | 3-5 |
|  | Granulation tissue | Connective tissue repair with new capillaries, surrounded by spindle-shaped fibroblasts, myofibroblasts | 4-5 |
|  | Irregular crypts | Non-parallel crypts, variable crypt diameters | 4-5 |
|  | Crypt loss | Mucosa devoid of crypts | 4-5 |
|  | Villous blunting | Villous-to-crypt-length ratio | 1-5 |

**Reference**

1. Erben, U. et al. A guide to histomorphological evaluation of intestinal inflammation in mouse models. *International Journal of Clinical and Experimental Pathology* **7**, 4557-U27 (2014).
